# Role of SIRT3 in bone homeostasis and its application in preventing and treating bone diseases

**DOI:** 10.3389/fphar.2023.1248507

**Published:** 2023-12-20

**Authors:** Ke Xu, Jing Li, Ruiming Wen, Bo Chang, Yang Cheng, Xuejie Yi

**Affiliations:** ^1^ School of Sports Health, Shenyang Sport University, Shenyang, China; ^2^ School of Physical Education, Liaoning Normal University, Dalian, China

**Keywords:** SIRT3, bone homeostasis, osteoblasts, osteoclasts, motility

## Abstract

Bone homeostasis refers to the balance between osteoblast-mediated bone formation and osteoclast-mediated bone resorption and the maintenance of stable bone mass. SIRT3 is a class of mitochondrial protein deacetylase that influences various mitochondrial functions and is involved in the mechanisms underlying resistance to aging; regulation of bone marrow mesenchymal stem cells, osteoblasts, and osteoclasts; and development of osteoporosis, osteoarthritis, and other bone diseases. Moreover, exercise affects bones through SIRT3. Thus, studies on SIRT3 may provide insights for the treatment of bone diseases. Although SIRT3 can exert multiple effects on bone, the specific mechanism by which it regulates bone homeostasis remains unclear. By evaluating the relevant literature, this review discusses the structure and function of SIRT3, reveals the role and associated mechanisms of SIRT3 in regulating bone homeostasis and mediating bone health during exercise, and highlights the potential pharmacological value of SIRT3 in treating bone diseases.

## 1 Introduction

Bone health strongly affects quality of life. The World Health Organization indicated that in 2021, approximately 1.71 billion cases of musculoskeletal disorders were reported worldwide, with over 300 million of these cases caused by osteoarthritis (OA) and 463 million caused by fractures^1^. Thus, bone-associated diseases exert a significant burden on healthcare systems worldwide, which necessitates improvements in prevention and treatment.

Bones maintain homeostasis through continuous bone formation and resorption (Harada and Rodan, 2003). During bone formation, bone marrow mesenchymal stem cells (BMSCs) differentiate into osteoblasts (OBs), which secrete collagen fibers and bone matrix, gradually accumulate calcium, and form bones (Mizoguchi and Ono, 2021); whereas during bone resorption, osteoclasts (OCs) dissolve and resorb the old bone formed via necrotic and apoptotic osteocytes (Da et al., 2021). The interaction between OBs and OCs maintains a normal healthy bone mass (Long, 2011; Croucher et al., 2016; Kalinkovich and Livshits, 2021).

The disruption of bone homeostasis results in several bone-associated diseases (Zaidi, 2007). Indeed, when the balance between OBs and OCs is disturbed, bone structure and function are impaired, ultimately resulting in bone diseases, such as osteoporosis (OP), OA, and intervertebral disc degeneration (IDD) (Feng and McDonald, 2011; Hügle and Geurts, 2017; Sun et al., 2021). Investigating the mechanisms underlying the imbalance in bone homeostasis provides insights into the prevention and treatment of bone diseases.

Sirtuin (SIRT) is a nicotinamide adenine dinucleotide (NAD+)-dependent histone deacetylase, and the SIRT family contains seven members: SIRT1–SIRT7 (Li et al., 2021b). SIRT3 is a longevity-related gene (McDonnell et al., 2015) that is primarily found in the mitochondria, and it can mitigate various aging-related diseases by promoting metabolism, maintaining metabolic homeostasis, alleviating oxidative stress, and delaying cell aging (Kincaid and Bossy-Wetzel, 2013; Zhang et al., 2020d). While previous studies have focused on mitochondrial SIRT3, glycolipid metabolism, and aging, Huh et al. (2016) found that in mice, SIRT3 is involved in maintaining bone homeostasis by regulating the AMP-activated protein kinase-peroxisome proliferator-activated receptor-γ co-activator-1β (AMPK-PGC-1β) axis (Huh et al., 2016). These findings facilitated subsequent studies on the role of SIRT3 in bone homeostasis-related diseases. SIRT3 plays important regulatory roles in the formation and resorption of bone (Gao et al., 2018; Li et al., 2021c) and the development of bone diseases, such as OP, OA, and IDD, by regulating mitochondrial function. For example, SIRT3 overexpression in adeno-associated viral vectors in SAMP6 mice (a model of senile osteoporosis (SOP)) showed that SIRT3 regulates advanced glycation end product (AGE)-induced SOP by modulating mitochondrial autophagy (Guo et al., 2021). Similarly, SIRT3 activators and inhibitors affect the development of OBs and OCs (Ling et al., 2021; Li et al., 2022c).

**FIGURE 1:**
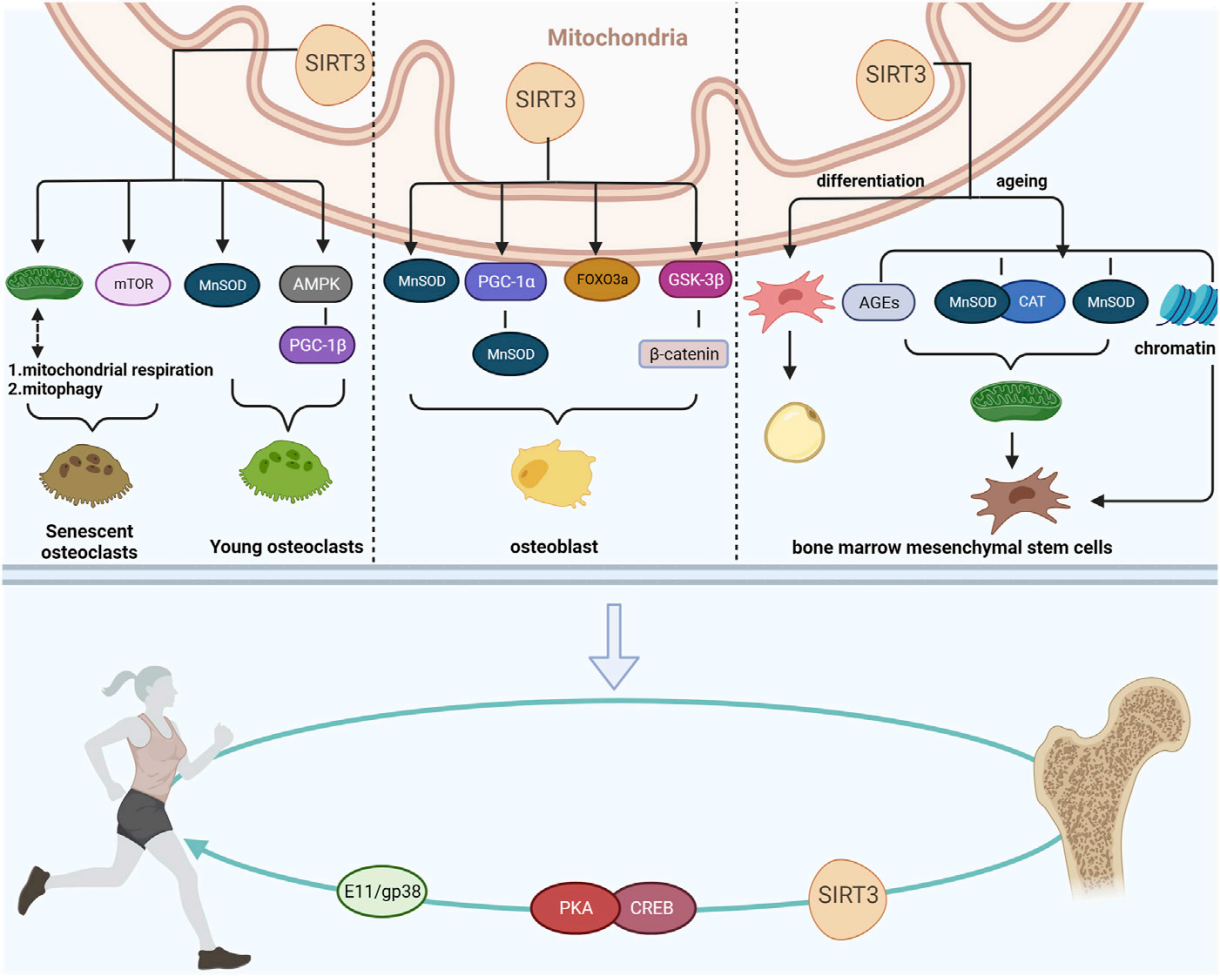
Mechanism via which SIRT3 maintains bone homeostasis and its relationship to exercise.

A previous study suggested that SIRT3 also appears to have a potential relationship with exercise, mediating exercise to regulate bone mass and improve bone disorders associated with bone homeostasis. (Li et al., 2022c). Therefore, we reviewed the roles and mechanisms of SIRT3 action in regulating bone homeostasis-related diseases to provide novel insights into the role of SIRT3 in preventing and treating bone homeostasis-related diseases (Figure 1).

## 2 Mechanisms for maintaining bone homeostasis

Although bones are inactive at the cellular level, OBs are constantly engaged in cellular metabolism and exchange, and various bone cells play important roles in maintaining bone homeostasis.

**FIGURE 2:**
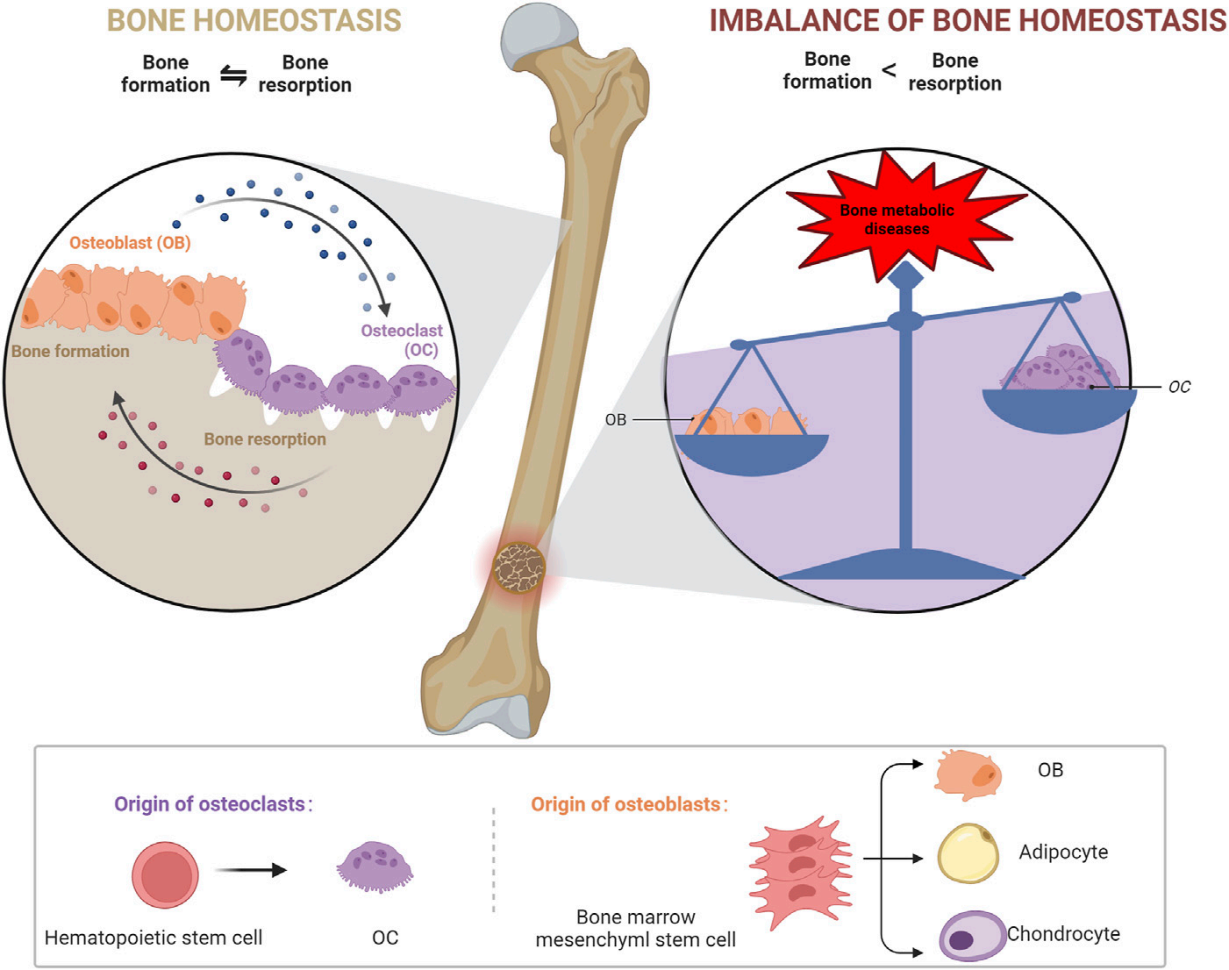
Mechanisms maintaining bone homeostasis.

BMSCs are tissue-specific cells located in the mesoderm that have multiple differentiation potentials; for example, they can differentiate into OBs, adipocytes, and chondrocytes (Kfoury and Scadden, 2015). OBs are primarily derived from BMSCs (Zhou et al., 2008) and promote bone matrix synthesis, secretion, and mineralization and regulate OC differentiation (Maruotti et al., 2017; Han et al., 2018). OCs are multinucleate cells derived from monocyte/macrophage lineage cells and are mainly located on in the bone matrix surface, and they degrade old bone tissue by secreting acids and protein hydrolases, such as histone K (Roodman, 1996). OBs, chondrocytes, and endothelial cells mediate bone homeostasis by regulating the activity of OBs and OCs, and bone homeostasis is driven by cross-talk between OBs and OCs; therefore, an imbalance between OBs and OCs interferes with bone tissue physiology and can lead to metabolic diseases, such as OP and osteosclerosis (Wang et al., 2020b). Therefore, the mechanisms underlying bone diseases can be better understood by elucidating bone homeostatic regulation via SIRT3 (Figure 2).

## 3 Structure and function of SIRT3

SIRT3 is a typical class I SIRT that is mainly expressed in mitochondria (Zhang et al., 2020d). SIRT3 proteins are classified into full-length and mature types. Full-length SIRT3 proteins are primarily found in the nucleus, contain 399 amino acids, and lack deacetylase activity. When full-length SIRT3 moves into the mitochondria, it is cleaved by mitochondrial matrix processing peptidases to form an active 28 kDa protein, i.e., mature SIRT3 (Wang et al., 2021). SIRT3 contains a conserved enzyme core region with two structural domains, namely a larger Rossmann folded structural domain bound to DNA+ and a smaller structural domain consisting of a complex helical structure and zinc-binding motif with the acetylated substrate bound in the gap between the two domains (Nguyen et al., 2013).

**FIGURE 3:**
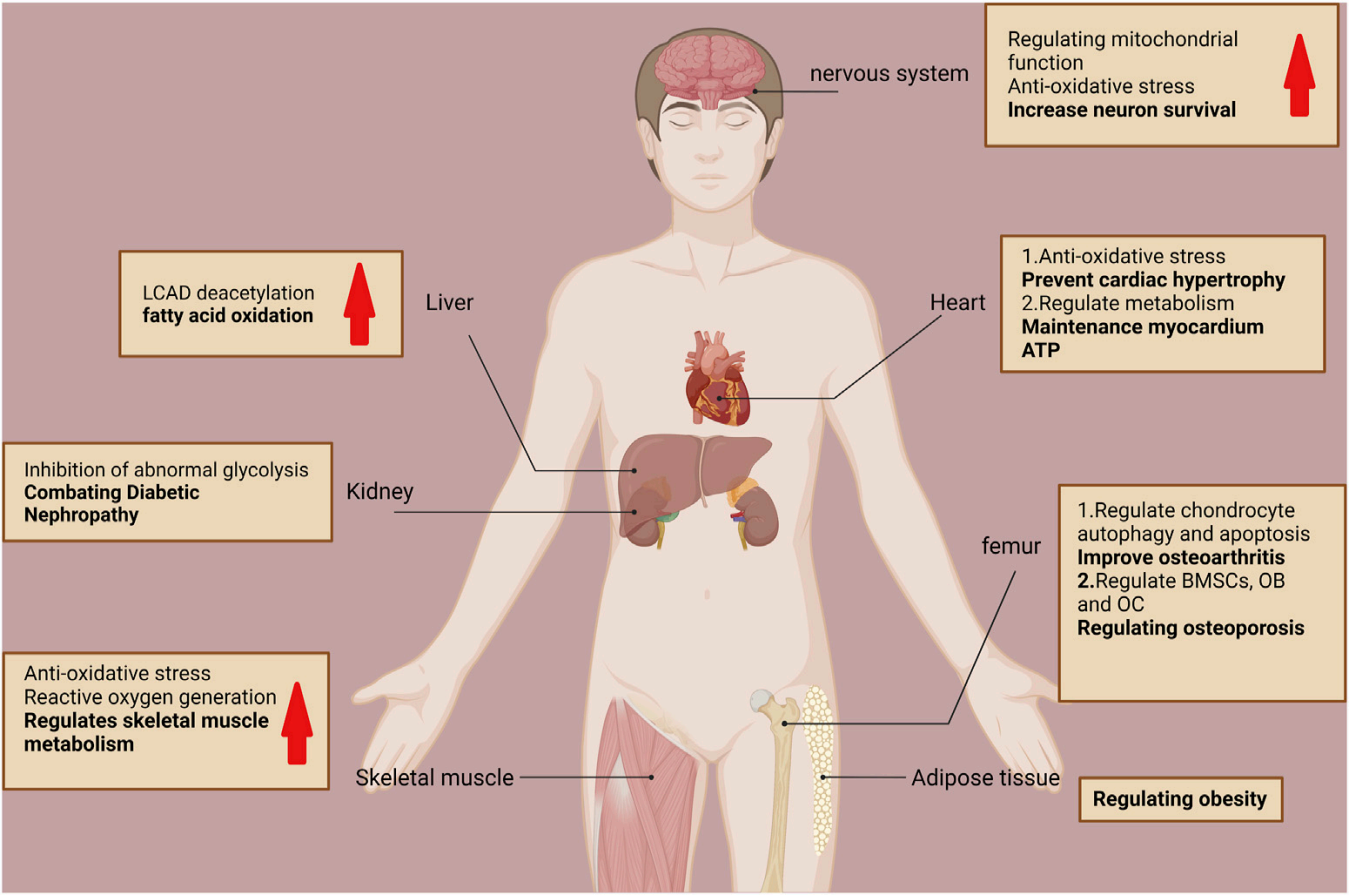
Role of SIRT3 in various organ tissues.

Notably, acetylation modifications influence all aspects of mitochondrial function and fate. SIRT3 is the predominant mitochondrial deacetylase that directly regulates the acetylation of at least 84 mitochondrial proteins (Yang et al., 2016). These proteins are involved in almost all mitochondrial functions, including mitochondrial mitophagy (CHCHD3 and IMMT), transcription (TFAM and MTIF2), translation (MRPL11 and MRPS34), DNA processing (POLB and POLDIP2), RNA processing (PNPT1 and RNMTL1), lipid metabolism (ACADM and AGK), ETC/OXPHOS (NDUFA5 and ATP5B), the TCA cycle (OGDH and DLST), and amino acid metabolism (GLUD1 and OAT) (Yang et al., 2016). SIRT3 promotes mitochondrial dynamic homeostasis mainly by regulating the acetylation levels of its substrates. SIRT3 regulates numerous cellular physiological and pathological processes by regulating mitochondrial homeostasis (e.g., oxidative stress inhibition), promoting energy metabolism, and regulating mitochondrial autophagy (He et al., 2020). For example, SIRT3 promotes energy metabolism by regulating long-chain acyl-coenzyme A dehydrogenase-induced fatty acid oxidation through its powerful deacetylation function, and systemic SIRT3 knockout mice exhibit impaired fatty acid oxidation during fasting (Hirschey et al., 2010). Therefore, SIRT3 is critical in mitochondrial homeostasis, and its abnormal function can cause mitochondrial dysfunction and various diseases. SIRT3 is mainly associated with aging and age-related diseases, such as neurodegenerative diseases, cardiovascular diseases, sarcopenia, metabolic syndrome, and diabetic nephropathy (Hirschey et al., 2011; Jing et al., 2011; Srivastava et al., 2018; Sun et al., 2018; Anamika et al., 2019). Oxidative stress triggered by mitochondrial dysfunction can also impair OB differentiation and increase OC activity, which can disrupt bone homeostasis and lead to a series of bone diseases (Domazetovic et al., 2017). SIRT3 can downregulate mitochondrial function and biogenesis through the PGC-1α/MnSOD signaling pathway when SIRT3 is knocked down, thereby impairing OB differentiation (Ding et al., 2017). SIRT3 also positively affects bone health by improving OA and resistance to OP (Xu et al., 2021; Hu and Wang, 2022) (Figure 3).

## 4 Mechanisms underlying SIRT3 regulation of bone homeostasis

### 4.1 Effects of SIRT3 on BMSCs

#### 4.1.1 Regulation of BMSCs differentiation by SIRT3

BMSCs can self-regenerate and exhibit multidirectional differentiation, and their tendency to differentiate into OBs and adipocytes determines the maintenance of bone and fat volume in the bone marrow cavity (Zhou et al., 2014a). Cell differentiation is regulated by differentiation factors induced in specific cell lineages. Therefore, regulating the osteogenic and lipogenic differentiation of BMSCs plays a crucial role in maintaining bone homeostasis.

With aging, the adipose tissue proportion in the bone marrow cavity gradually increases while bone mass decreases due to the increased adipogenic differentiation ability of BMSCs and the decrease in osteogenic differentiation (Ambrosi et al., 2017). In general, a reciprocal relationship is observed between adipogenic and osteogenic differentiation of BMSCs, and its regulation is facilitated by SIRT3.

After transfecting third-generation BMSCs with retroviral vectors targeting shRNA for SIRT3, both the lipogenic and osteogenic differentiation of BMSCs was significantly reduced when SIRT3 was knocked down, while oxidative stress was alleviated and the differentiation capacity and lifespan of aged BMSCs (generation 7) were restored when SIRT3 was overexpressed (Denu, 2017). This suggests that aging-related defects can be mitigated by increasing SIRT3 expression in later-passaged BMSCs, which may represent another emerging mechanism by which SIRT3 regulates aging-related bone disease.

Ho et al. (2017) established a SIRT3-overexpression mouse model and observed that in 13-month-old males, adipocyte numbers in the longitudinal tibial sections were significantly higher, lipogenic differentiation was significantly enhanced, and osteogenic differentiation was inhibited in cultured BMSCs (Ho et al., 2017). This suggests that SIRT3 overexpression in BMSCs promotes adipogenic differentiation in senescent male mice. In the control experiments, the authors observed no skeletal regulation by SIRT3 in 13-month-old female mice and 6-month-old male mice (Ho et al., 2017). Therefore, further investigations are required to determine the effects of SIRT3 on the osteogenic and lipogenic differentiation of BMSCs in young mice.

**FIGURE 4:**
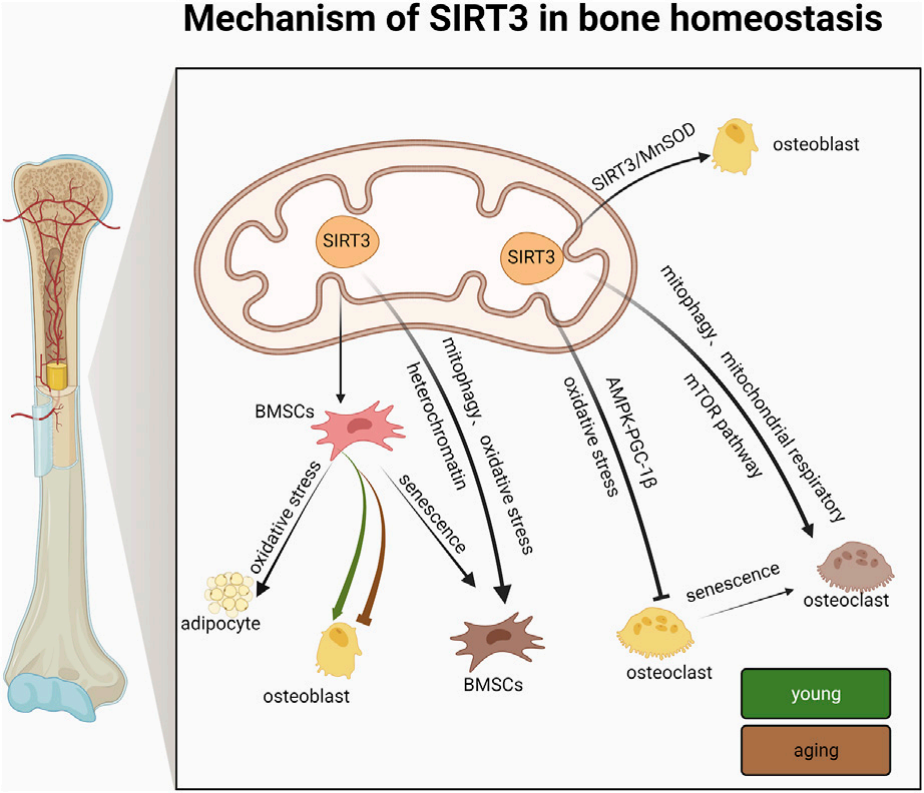
Mechanism of action of SIRT3 in bone homeostasis.

With regard to differentiation capacity, studies have shown conflicting results. Some studies have shown that the differentiation ability of BMSCs generally decreases with age (Asumda and Chase, 2012); On the other hand, some studies have the idea of “adipogenic switch”, that is, with the progress of aging, the osteogenic differentiation ability of BMSCs decreases, and the adipogenic differentiation ability increases (Moerman et al., 2004). These two views are consistent with the two experimental results described above. SIRT3 appears to have extremely complex regulatory roles in BMSC differentiation and bone homeostasis that may be correlated with age and sex. Therefore, the differences in BMSC differentiation regulated by SIRT3 and the specific underlying mechanisms should be further investigated in terms of age and sex (Figure 4; Table 1).

**TABLE 1:** Role of SIRT3 in BMSCs.

Osteogenic and lipogenic differentiation of BMSCs				BMSCs ageing			
Expression	Pathways	Function	References	Expression	Pathways	Function	References
Down	Anti- oxidative stress	All lower	Denu (2017)	Up	Activation of mitochondrial autophagy	Anti-aging of BMSCs	Guo et al. (2021)
					Genistein/ERRα/		Li et al. (2023a)
					SIRT3		
Up		Enhanced lipogenic differentiation (senescent mice)	Ho et al. (2017)		SIRT3/MnSOD		Wang et al. (2014), Ma et al. (2020)
					Foxo3a/MnSOD&CAT		Zhang et al., 2018; 2020a
					Stabilization of heterochromatin		Diao et al. (2021)
					S-sulfhydration/SIRT3/RUNX2/PPARγ		Liu et al. (2023)

#### 4.1.2 Role and mechanism of action of SIRT3 in BMSC aging

BMSC senescence plays an important role in bone homeostasis. Along with aging, BMSCs show skeletal senescence characteristics after exposure to various endogenous and exogenous senescence factors, including mitochondrial autophagy, oxidative stress, chromatin organization distortion, and DNA damage. Such characteristics lead to the abnormal lipogenic/osteogenic differentiation of BMSCs, which disrupts bone homeostasis and decreases bone mass (Qadir et al., 2020).

SIRT3 has been shown to regulate mitochondrial autophagy via the Forkhead box O3a/PTEN-inducible putative kinase protein 1 (Foxo3a/PINK1)-Parkin signaling pathway, which promotes diabetic corneal epithelial wound healing *in vivo* and *in vitro* and improves diabetic keratopathy (Hu et al., 2019). Mitochondrial autophagy is closely related to aging (Guo and Chiang, 2022). AGEs promote BMSC aging by inhibiting mitochondrial autophagy (Guo et al., 2021). Notably, the overexpression of SIRT3 in SAMP6 mice (SOP model) reverses AGE-induced senescence in BMSCs, while silencing SIRT3 exacerbates this effect (Guo et al., 2021). These findings suggest that SIRT3 can reverse AGE-induced senescence in BMSCs by activating mitochondrial autophagy and improving mitochondrial function.

Improving mitochondrial autophagy by targeting SIRT3 is a potential therapeutic strategy to attenuate AGE-associated SOP (Guo et al., 2021). SIRT3 directly deacetylates and activates PINK1, which recruits Parkin from the cytoplasm to the mitochondria, ubiquitinates mitochondrial outer membrane proteins, and ultimately achieves mitochondrial autophagy (Wang et al., 2020a; Ling et al., 2021; Li et al., 2022a). ERRα binds to the proximal SIRT3 gene promoter region to promote SIRT3 expression (Giralt et al., 2011). Li et al. (2023a) found that in ovariectomized rats, genistein mitigates the upregulation of SIRT3 expression via ERRα, promotes mitochondrial biogenesis, and reduces mitochondrial autophagy, which in turn alleviates senescence in BMSCs (Li et al., 2023a). In contrast, ERRα siRNA treatment significantly inhibits SIRT3 expression. SIRT3 is involved in the positive effects of ERRα on BMSC aging by regulating mitochondrial biogenesis and autophagy. Although the above findings suggest that SIRT3-based regulation of mitochondrial autophagy may be a mechanism of aging and senile OP in BMSCs, the specific mechanism and changes in the related signaling pathways remain to be explored. Further research into the relationship among SIRT3, aging, and mitochondrial autophagy in BMSCs will provide novel insights.

SIRT3 is a key oxidative stress regulator that protects cardiomyocytes from stress-mediated apoptosis through Ku70 deacetylation (Sundaresan et al., 2008). However, Ma et al. (2020) compared age-associated natural senescence and a rat model of hydrogen peroxide (H_2_O_2_)-induced premature senescence in BMSCs and found that the expression of SIRT3 was significantly reduced in both models and closely associated with changes in the cellular reactive oxygen species (ROS) levels and increased DNA damage. SIRT3 supplementation partially reverses the senescence-related phenotypic features of both naturally and prematurely senescent BMSCs. SIRT3 ameliorates oxidative stress damage by upregulating the expression and activity of manganese superoxide dismutase (MnSOD) and reducing ROS levels (Ma et al., 2020). Similarly, SIRT3 overexpression enhances the anti-oxidative stress activity of BMSCs by activating MnSOD and catalase, thereby increasing the cell survival rate (Wang et al., 2014). In addition, SIRT3 can positively regulate catalase and MnSOD by translocating FOXO3a into the nucleus, thereby protecting aged BMSCs from oxidative stress injury (Zhang et al., 2018; Zhang et al., 2020a.). The above results indicate that SIRT3 delays the aging of BMSCs through antioxidant effects and reveal that MnSOD is an important linking factor in the regulation of aging in BMSCs by SIRT3. Further mechanistic studies are warranted to explore the close connection between the two, which may provide deeper insights into the treatment of aging-related metabolic bone diseases.

Chromatin abnormalities are important epigenetic alterations that contribute to aging (Saul and Kosinsky, 2021). However, data on the association of SIRT3 with chromatin abnormalities are limited. SIRT3 interacts with nuclear lamins and heterochromatin-associated proteins to promote heterochromatin structural stabilization, and CRISPR/cas9-mediated deletion of SIRT3 impairs the nuclear integrity of human mesenchymal stem cells (hMSCs), causes heterochromatin loss, and accelerates senescence (Diao et al., 2021). Supplementing SIRT3 in SIRT3-deficient hMSCs attenuates chromatin loss and hMSC senescence, suggesting that SIRT3 may protect aged hMSCs partly by stabilizing heterochromatin (Diao et al., 2021). In addition, the S-sulfhydration of cysteine residues enhances SIRT3 activity through the formation of persulfides and the exogenous hydrogen sulfide (H_2_S) donor NaHS-mediated enhancement of SIRT3 rescues the senescent phenotype of BMSCs (Liu et al., 2023). Conversely, SIRT3 deficiency accelerates oxidative stress-induced senescence in BMSCs by increasing mitochondrial dysfunction and heterochromatin instability.

Moreover, H_2_S-mediated S-sulfhydration modification ameliorates dithiothreitol (S-sulfhydration inhibitor)-induced heterochromatin abnormalities and mitochondrial fragmentation, thereby promoting osteogenic differentiation and inhibiting BMSC senescence (Liu et al., 2023).

However, a mutation in the CXXC position of the SIRT3 zinc finger motif eliminates the anti-aging effect of S-sulfhydration modification on BMSCs, which suggests that the effect of the S-sulfhydration modification is closely related to SIRT3. A more in-depth mechanistic study revealed that S-sulfhydration regulates the lipogenic/osteogenic differentiation of BMSCs through the SIRT3/RUNX2/PPARγ axis (Liu et al., 2023). This finding showed for the first time that H_2_S-mediated S-sulfhydration is a novel post-translational modification of SIRT3 that counteracts senescence in BMSCs by modulating mitochondrial and heterochromatin homeostasis, thereby providing novel insights into restoring the senescence-induced failure of BMSCs. However, whether an intrinsic link occurs between mitochondrial homeostasis and heterochromatin mediated by SIRT3 S-sulfhydration remains to be determined.

In summary, SIRT3 mainly affects BMSC differentiation and senescence. Under normal conditions, SIRT3 promotes the conversion of BMSCs to adipocytes, which adversely affects BMSCs, whereas under aging conditions, SIRT3 delays the conversion of BMSCs by activating mitochondrial autophagy, inhibiting oxidative stress, and consolidating chromatin stability. Unlike previous studies that attributed SIRT3-based regulation of cellular senescence to the regulation of mitochondrial function, the above studies suggest that SIRT3 may also resist BMSC senescence by consolidating heterochromatin stability. These new findings not only expand our understanding of the epigenetic regulation of heterochromatin during human cellular senescence but also provide deeper insights into the mechanisms by which SIRT3 regulates the mechanism underlying BMSC senescence, thus providing potential drug targets for developing and preventing novel therapeutic strategies for bone diseases associated with bone homeostasis (Figure 4; Table 1).

### 4.2 Role of SIRT3 in OBs

Osteogenic differentiation is key in OB-mediated bone formation and essential for bone homeostasis. Mitochondrial dysfunction is the underlying pathological cause of dysfunctional osteogenic differentiation; therefore, maintaining normal mitochondrial function and biogenic homeostasis is essential for osteogenic differentiation (Tsai et al., 2015). SIRT3 expression and deacetylase activity are essential for the osteogenic differentiation of the mouse pre-osteogenic cell line MC3T3-E1. After infecting MC3T3-E1 with a lentivirus carrying SIRT3 and stabilizing and knocking down the SIRT3, SIRT3 deletion significantly reduces the peroxisome proliferator-activated receptor-γ co-activator-1ɑ (PGC-1α)/MnSOD signaling pathway-mediated reduction in mitochondrial function and biogenesis, thereby inhibiting osteogenic differentiation (Ding et al., 2017). Meanwhile, MnSOD can be specifically induced to eliminate excess mitochondrial superoxide and protein oxidation during OB differentiation, whereas increased SIRT3 expression enhances MnSOD activity by deacetylating K68 (Gao et al., 2018). The knockdown of MnSOD and SIRT3 inhibits OB differentiation. SIRT3-deficient mice exhibit significant bone loss and OB dysfunction, whereas the overexpression of either MnSOD or SIRT3 enhances primary OB differentiation in SIRT3-deficient mice (Gao et al., 2018). These findings suggest that the SIRT3/MnSOD axis plays a crucial role in OB differentiation by regulating mitochondrial function and that activating SIRT3 is a potential therapeutic strategy for treating related bone diseases. SIRT3/MnSOD plays an important role in BMSC senescence and is highly relevant to OB. Further investigations are warranted to explore the mechanism of SIRT3/MnSOD activation and determine the molecular correlation between OB and OC.

In addition to MnSOD, there are also some external factors that can affect SIRT3 expression. Nicotinamide (NAM), the water-soluble form of vitamin B3, is a food additive with a favorable safety profile in clinical trials (Chen et al., 2015). As one of the precursors of nicotinamide adenine dinucleotide (NAD), NAM induces the activation of SIRTs (e.g., SIRT1 and SIRT3) and transcriptional regulators (e.g., PGC1A and FOXO) by increasing the NAD level and promoting mitochondrial biogenesis and functions (Srivastava, 2016). *Ex vivo*, NAM also alleviates mitochondrial ROS levels and stimulates OB differentiation by enhancing the expression and activity of mitochondrial antioxidant enzymes (Yoon et al., 2023). In MC3T3-E1 cells, NAM has been shown to dose-dependently increase SIRT3 activity and activate FOXO3a through SIRT3, which in turn promotes the expression of mitochondrial antioxidant enzymes, ultimately promoting OB differentiation (Yoon et al., 2023). These results suggest that NAM may represent a therapeutic or preventive agent to improve bone health, and SIRT3 undoubtedly plays a crucial role. The upstream and downstream factors of SIRT3 that regulate bone health and their mechanisms should be further explored to provide a development direction for new clinical drugs.

Nicotine is the main addictive agent in tobacco products and represents a risk factor for various diseases; moreover, it regulates OB differentiation *in vitro*, thus affecting bone metabolism (Kim et al., 2012). Nicotine significantly reduces MnSOD activity by decreasing SIRT3 levels, which leads to mitochondrial oxidative stress and DNA damage in OBs. In contrast, high SIRT3 expression upregulates MnSOD activity by deacetylating MnSOD. These findings indicate that the SIRT3-MnSOD axis is affected by nicotine and increased SIRT3 expression alleviates nicotine-induced mitochondrial oxidative stress and mitochondrial DNA damage in OBs (Li et al., 2015).

Prolonged exposure to microgravity in space or on space stations can pose numerous hazards to the musculoskeletal system. Based on the statistics, continuous exposure to microgravity can result in a monthly bone loss of up to 1%, with an average decrease in skeletal muscle strength of 30% (Williams et al., 2009; Willey et al., 2011). Consequently, reducing the musculoskeletal system damage caused by exposure to microgravity is of substantial importance for promoting the development of space exploration. Moreover, recent studies have determined that activating SIRT3 induces mitochondrial autophagy in myoblasts (C2C12) and reduces C2C12 death induced by microgravity, maintaining the expression of C2C12 differentiation markers (Aventaggiato et al., 2023), which suggests that activating SIRT3 may reduce muscle tissue damage caused by microgravity. Notably, muscles and bones are closely related, and together, they constitute the musculoskeletal regulatory system, integrating relevant biological functions. As mentioned earlier, mitochondrial autophagy also has a remarkable effect in promoting osteoblast differentiation. Consequently, considering the crucial role of SIRT3 in bone formation, investigating whether activating SIRT3 can also mitigate the hazards of microgravity in the skeletal system is highly valuable. Simultaneously, this suggests that activating SIRT3 could represent a targeted molecular strategy. Subsequent investigations will be required to identify external agents or pharmaceuticals capable of stimulating SIRT3 expression, thereby facilitating the development of novel strategies to enhance bone health.

Peripheral prosthetic osteolysis is the main factor underlying implant failure in arthroplasty, which occurs via titanium particle wear (Goodman, 2007). Titanium ions induce the overproduction of mitochondrial ROS, which damage OBs (Wang et al., 2020c) and is particularly important in counteracting oxidative stress and promoting osteogenic differentiation. The upregulation of SIRT3 can improve periprosthetic osteolysis by inhibiting NLRP3 inflammatory vesicles, promoting osteogenesis via the glycogen synthase kinase-3β (GSK-3β)/β-connexin signaling pathway, and improving titanium particle-induced osteogenesis inhibition (Zheng et al., 2021). These results suggest that SIRT3 is a positive regulator of osteogenic and OB differentiation. SIRT3/MnSOD is a potential target for promoting bone health and treating bone diseases related to bone homeostasis (Figure 4; Table 2).

**TABLE 2:** Role of SIRT3 in the OB.

Expression	Pathways	Function	References
Down	PGC-1α/MnSOD	Inhibition of osteogenic differentiation	Ding et al. (2017)
Down	SIRT3/MnSOD	Inhibition of osteogenic differentiation	Gao et al. (2018)
Up	SIRT3/FOXO3a	Promotes osteogenic differentiation	Yoon et al. (2023)
Up	SIRT3/MnSOD	Alleviation of oxidative stress in OB mitochondria	Li et al. (2015)
Up	GSK-3β/β-linked protein	Promotes osteogenic differentiation	Zheng et al. (2021)

### 4.3 Role of SIRT3 in OCs

Human OCs are unique bone-resorbing cells with an important role in normal bone transformation, regeneration, and remodeling. Damage to OC activity may progressively reduce bone regeneration capacity with age; thus, strict regulation of OC formation is essential for bone homeostasis (McArdle et al., 2015). The effect of SIRT3 on OCs is complex and likely age-related.

SIRT3 contributes to increased OC bone resorption during skeletal aging. Ling et al. investigated the role of SIRT3 in age-associated bone loss in mice and found that 16-month-old SIRT3 systemic knockout (KO) mice had significantly greater cortical thickness than WT controls of the same age (Ling et al., 2021). Serum from these SIRT3 KO mice had significantly reduced levels of c-terminal peptide of type 1 collagen (bone resorption marker), suggesting that the high bone mass phenotype of SIRT3 KO aged mice is at least partially due to OC-mediated bone resorption in SIRT3 KO mice. Moreover, following the isolation of primary bone marrow-derived macrophages (BMDMs) from SIRT3 KO senescent mice and induction of osteoclastogenesis, the OCs of KO mice were significantly smaller than those of WT mice, suggesting impaired OC function (Ling et al., 2021).

Mechanistic studies have shown that SIRT3 deficiency can impair bone resorption by attenuating mitochondrial respiration and mitosis in OCs (Ling et al., 2021). This finding highlights a promising strategy for treating age-related bone diseases via the manipulation of SIRT3 expression in OCs to regulate the dynamic homeostasis of bone. It also reconfirms the contribution of SIRT3-regulated mitochondrial function to bone diseases. Similarly, overexpression of SIRT3 during the aging stage has a positive effect on promoting bone resorption. For example, Ho et al. found that the bone mass of 13-month-old SIRT3-overexpressing mice was significantly reduced compared with that of control mice (Ho et al., 2017) while the number of tartrate resistant acid phosphatase-positive cells/bone surface increased by 2.5-fold compared with that of the controls. These findings further support the notion that SIRT3 regulates bone mass in part through OC-mediated bone resorption. More in-depth mechanistic studies have shown that SIRT3 primarily activates the mammalian target of rapamycin (mTOR) pathway to positively affect OC formation (Ho et al., 2017). Collectively, these results suggest that SIRT3 enhances osteogenesis during senescence and promotes OC and bone resorption functions.

Notably, SIRT3 negatively regulates OC differentiation, and its systemic knockdown significantly enhances OC differentiation in 8-week-old wild mice, which causes a marked reduction in bone mass (Huh et al., 2016). Moreover, following the induction of OC formation via BMDM treatment with receptor activator of nuclear factor-κB ligand (RANKL), the overexpression of PGC-1β significantly increases the expression of SIRT3 mRNA and protein levels. Similarly, PGC-1β knockdown downregulates SIRT3 expression levels, suggesting that RANKL induces pGC-1β expression by increasing SIRT3 at the transcriptional level (Huh et al., 2016). In addition, the siRNA-mediated KO of SIRT3 in BMDMs results in a significant decrease in the total protein and phosphorylation levels of AMPKα. Following the overexpression of AMPKα in SIRT3-KO BMDMs, OC production becomes significantly reduced compared to that of the controls (Huh et al., 2016), suggesting that SIRT3 negatively controls OC differentiation by regulating AMPKα protein expression at the protein level. Thus, SIRT3 appears to function as a bridge between PGC-1β and AMPK to regulate OC differentiation via the AMPK-PGC-1β pathway (Huh et al., 2016).

ROS are indispensable secondary messengers in RANKL-induced OC differentiation and activation (Kim et al., 2017). SIRT3 inhibits OC differentiation by enhancing MnSOD activity via deacetylating lysine 68 of MnSOD and reducing the ROS level in OCs (Kim et al., 2017). Meanwhile, SIRT3 knockdown increases OC production in 5-week-old female Institute of Cancer Research mice and significantly enhances RANKL-induced bone loss (Kim et al., 2017). Thus, the role of SIRT3 in young mice appears to be inconsistent with that in aging mice; that is, SIRT3 may negatively regulate OC differentiation and bone mass at a young age. These two conflicting results suggest that the effect of SIRT3 on OCs is age-dependent. However, such studies are limited because of the lack of a detailed definition of the age-dependence of SIRT3-induced OC regulation, i.e., when SIRT3 shifts from negative to positive regulation. This important issue should be addressed in future studies.

Furthermore, SIRT3 does not affect OC function via a single pathway. Ionizing radiation increases SIRT3 activity and mitochondrial respiration in OCs, thereby promoting OC differentiation and bone loss in adult male mice. However, in the absence of SIRT3, bone resorption is not induced in OCs, suggesting that SIRT3 is responsible for this process (Richardson et al., 2022). Hence, targeting OC mitochondrial activity may be a novel strategy for treating radiation-induced bone loss, which is likely mediated primarily by SIRT3. SIRT3 inhibition can also prevent titanium particle-induced bone resorption and OC production by inhibiting extracellular signal-regulated kinases and c-Jun N-terminal kinase signaling, thus indicating a new avenue for combatting periprosthetic osteolysis (Li et al., 2021a). Therefore, the specific mechanism of action employed by SIRT3 in regulating OC differentiation and bone mass in mice of different ages warrants further investigation (Figure 4; Table 3).

**TABLE 3:** Role of SIRT3 in OC.

	Expression	Pathways	Function	References
Aging	Down	Attenuates mitochondrial respiration and autophagy	Impaired bone resorption	Ling et al. (2021)
	Up	Activate mTOR	Promoting OC generation	Ho et al. (2017)
Young	Down	AMPK-PGC-1β	Promoting OC differentiation	Huh et al. (2016)
	Up	Oxidative stress, mitochondrial respiration and autophagy	Inhibition of OC differentiation	Kim et al. (2017)

In summary, SIRT3 can regulate bone homeostasis by affecting the activity of BMSCs, OBs, and OCs. However, the precise mechanism remains unclear, and more in-depth studies are needed to provide a theoretical foundation for clinically treating bone homeostasis-related diseases.

## 5 Importance of SIRT3 in preventing and treating bone diseases

Increasing evidence has shown that SIRT3 has a unique role in maintaining human bone metabolism and homeostasis and preventing bone diseases.

### 5.1 Osteoporosis

OP is a typical aging-related disease (Lane et al., 2000) common in middle-aged and older adults, and it is characterized by decreased bone density and quality, deteriorated bone microarchitecture, and increased bone fragility and susceptibility to fracture. As such, OP is a public health issue that greatly impacts quality of life. SIRT3, as a longevity factor, is closely associated with aging and represents a potential target for treating OP (Hu and Wang, 2022). Moreover, aging-induced accumulation of AGEs can significantly reduce bone density and mineralization (Tabara et al., 2019) and is a key mediator of OP pathogenesis. Notably, a SAMP6 mouse model showed that SIRT3 overexpression via intravenous injection of recombinant adeno-associated virus 9 carrying SIRT3 (rAAV-SIRT3) can effectively alleviate age-induced senescence and enhance the osteogenic differentiation of BMSCs to inhibit OP by activating mitophagy (Guo et al., 2021). This suggests that targeting SIRT3 to enhance mitophagy is a potential therapeutic strategy for alleviating age-related OP. Consistent with this, *in situ* transplantation of ovariectomized-induced OP model mice with BMSCs into the bone marrow cavity ameliorates bone loss (Liu et al., 2023). Notably, SIRT3 silencing significantly reduces this effect and the BMSC osteogenic differentiation potential. Hematoxylin and eosin staining confirmed the significant deposition of adipocytes in the bone marrow cavity of the ovariectomized group (Liu et al., 2023). Collectively, these findings suggest that SIRT3 helps to promote the osteogenic differentiation of BMSCs by preventing bone loss in osteoporotic mice.

Numerous drugs are available for OP prevention and treatment. Among them, zoledronic acid is a recognized bisphosphonate anti-OP drug that affects bone metabolism by inhibiting bone resorption, and it is clinically effective in both post-menopausal women with OP and SOP (Wang et al., 2022). Mechanistically, zoledronic acid has a protective effect on H_2_O_2_-induced SIRT3 down-regulation and MnSOD upregulation in BMSCs, whereas the silencing of SIRT3 blocks the protective effect of zoledronic acid on BMSCs. The associated mechanism of action might involve zoledronic acid-induced inhibition of oxidative stress through the SIRT3/MnSOD pathway, which accelerates the osteogenic differentiation of BMSCs and ultimately alleviates OP (Gianni, 2020).

Daphnetin (7,8-dihydroxycoumarin) is an active ingredient extracted from the genus *Raffinia* that inhibits RANKL-induced OC production *in vitro* and improves the progression of glucocorticoid-induced OP by activating Wnt/GSK-3β/β-catenin signaling (Liu et al., 2019; Wang et al., 2020d). In a recent study, mice were suspended from the hind limbs to establish a model of wasting OP, and subsequent administration of daphnetin maintained mitochondrial homeostasis by restoring the expression of SIRT3 and upregulating MnSOD to alleviate ROS in OCs, thus inhibiting OC differentiation and leading to decreased bone resorption. This suggests that daphnetin may inhibit bone resorption through the SIRT3/MnSOD pathway, thereby improving the progression of wasting OP, which may be useful for the clinical treatment of bedridden patients (Gao et al., 2022).

Metformin is a widely used hypoglycemic drug that protects bone health by reducing bone loss in ovariectomized mice by inhibiting E2F1-mediated autophagy of OC precursors^2^. Meanwhile, the targeted knockdown of SIRT3 in OBs treated with H_2_O_2_ and metformin inhibits the protective effect of metformin on OBs and increases their apoptosis (Yang et al., 2021). Mechanistically, metformin enhances SIRT3 expression via the phosphatidylinositol-3-kinase/protein kinase B (PI3K/AKT) signaling pathway to reverse H_2_O_2_-induced OB apoptosis (Yang et al., 2021).

Trehalose is a typical stress metabolite that regulates extracellular signal-regulated kinase phosphorylation by increasing cellular autophagy to inhibit OB-mediated osteoclast formation and alleviate the bone loss associated with primary biliary cirrhosis (Xu et al., 2020). Moreover, trehalose is involved in lipid metabolism processes that inhibit adipocyte hypertrophy (Arai et al., 2020). OB apoptosis increases following the induction of a high-fat state with palmitic acid (Cao et al., 2022), while palmitate-induced OB apoptosis is alleviated by the enhancement of autophagy with trehalose. Interestingly, the positive effect of palmitic acid on OB is counteracted by silencing SIRT3 gene expression (Cao et al., 2022), suggesting that high-fat-induced OB apoptosis may be related to SIRT3 expression. In more detail, trehalose prevents palmitic acid-induced OB apoptosis by upregulating SIRT3 and enhancing autophagy through the AMPK/mTOR/ULK3 signaling pathway (Cao et al., 2022), thus indicating a new strategy for treating high-fat-induced OP. Although most studies have explored the regulatory role of SIRT3 on age-related bone loss, this finding indicates that SIRT3 may also have a unique role in obesity-induced OP. Hence, investigating the specific mechanism underlying the regulatory role of SIRT3 in OP is necessary to provide new insights for the clinical improvement of OP.

Melatonin is an indoleamine secreted by the pineal gland that has antioxidant properties and may improve OP by promoting bone formation and inhibiting OC production (Wang et al., 2019; MacDonald et al., 2021). Melatonin can mitigate mitochondrial oxidative stress through the SIRT3/MnSOD signaling pathway, which promotes osteogenesis to increase the periprosthetic bone mass and reduces the risk of periprosthetic bone loss in patients in post menopause and with OP (Zhou et al., 2019). Melatonin also inhibits oxidative damage in osteogenic precursor cells and promotes osteogenesis by activating SIRT1, which regulates SIRT3 activity and inhibits p3Shc expression (Liu et al., 2022). These pathways have the potential for development as a treatment for OP.

In conclusion, SIRT3 not only directly impacts OP but is also activated by various drugs to regulate OP. In the future, studies exploring the mechanism of SIRT3 in regulating OP should assess the feasibility of combining SIRT3 with other anti-OP drugs to counteract the possible efficacy limitations of monotherapy.

### 5.2 Degenerative intervertebral disc disease

IDD is another age-related bone and joint disease in which oxidative stress-induced apoptosis of nucleus pulposus cells (NPCs) may play a prominent role (Chen et al., 2016). Oxidative stress leads to dysfunctional bone homeostasis (Zhu et al., 2022a). SIRT3 is a key regulator of oxidative stress that also delays IDD progression by improving mitochondrial redox homeostasis (Zhang et al., 2020c). Lin et al. found that overexpressing SIRT3 significantly inhibits oxidative stress and extracellular matrix degradation in NPCs, thereby delaying their senescence and reducing IDD (Lin et al., 2021). The AMPK/PGC-1α pathway mediates the SIRT3 anti-oxidative stress-induced delay of NPC senescence (Lin et al., 2021). Similarly, SIRT3 knockdown in rat NPCs significantly reduces the tolerance of NPCs to oxidative stress. Notably, activating SIRT3 with honokiol (HKL) also ameliorates IDD in rats via the AMPK/PGC-1α/SIRT3 signaling pathway^3^. SIRT3 expression is also reduced in degenerative NP, and a study implementing a 3-month-old mouse model of IDD showed that the overexpression of SIRT3 delays IDD by activating the SIRT3/FOXO3/MnSOD signaling pathway to counteract oxidative stress (gianni, 2019). Collectively, these findings suggest that SIRT3 is closely related to IDD and its activators may inform the application of existing drugs that can prevent IDD.

SIRT3 also ameliorates IDD through alternative mechanisms. For instance, heat shock protein (Hsp70), a ubiquitous molecular chaperone, inhibits mitochondrial fission by upregulating SIRT3 expression, which attenuates compression-induced NPC apoptosis (Hu et al., 2021). Notably, nicotinamide mononucleotide, a biologically active nucleotide in clinical trials, restores SIRT3 function and reduces apoptosis through the AMPK/PGC-1α signaling pathway in human NPCs induced by AGES (Song et al., 2018). This reaffirms that SIRT3 may be a valuable candidate target for preventing and treating IDD.

### 5.3 Osteoarthritis

OA is another common bone and joint disease characterized by chondrocyte senescence and apoptosis, extracellular matrix degradation accompanied by synovial inflammation, and subchondral bone dysfunction (Malemud, 2017). The excessive production of ROS impairs chondrocyte metabolic homeostasis (Khan et al., 2017). Compared with normal chondrocytes, those in OA have impaired mitochondrial membrane potential and reduced complex I, II, and III activity, suggesting that mitochondrial dysfunction is involved in chondrocyte degeneration (Maneiro et al., 2003). Therefore, targeting mitochondrial homeostasis and enriching therapeutic approaches for OA represent promising strategies. As an important regulator of mitochondrial and bone homeostasis, SIRT3 represents a likely starting point.

SIRT3 can inhibit oxidative stress and improve the integrity and function of mitochondrial DNA in OA chondrocytes by activating AMPK; thus, SIRT3 may represent another emerging mechanism for AMPK protection of chondrocytes against OA (Chen et al., 2018). SIRT3 regulates chondrocyte autophagy and apoptosis through the PI3K/Akt/mTOR pathway to improve OA (Xu et al., 2021). However, SIRT3 also promotes high-fat-induced OA by inhibiting glycolysis and stimulating mitochondrial respiration and fatty acid metabolism (Zhu et al., 2022b). In contrast, SIRT3 deficiency in cartilage under high-fat conditions has a protective effect on cartilage at a young age, whereas the systemic deficiency of SIRT3 accompanied by aging accelerates OA progression (Fu et al., 2016). These results suggest that the role of SIRT3 in cartilage homeostasis is likely highly dependent on the environment and similar to its role in bone homeostasis because both are cell-specific and age-dependent.

In addition, the pathways by which SIRT3 regulates OA are diverse, with multiple classes of factors that regulate mitochondrial homeostasis and modulate OA progression by regulating SIRT3. For example, dihydromyricetin, also known as aminobenzene protease, has anti-inflammatory, antioxidant, and antitumor properties and inhibits oxidative stress by upregulating SIRT3 levels under low-pressure hypoxic conditions to exert a neuroprotective effect. As such, dihydromyricetin is a potential agonist of SIRT3 (Zhou et al., 2014b; Liu et al., 2016). Moreover, although SIRT3 abundance decreases with cartilage degeneration, dihydromyricetin can activate SIRT3-mediated mitochondrial homeostasis and reverse chondrocyte degeneration (Wang et al., 2018). This provides an emerging target for SIRT3 in OA treatment and further enriches the upstream and downstream factors of SIRT3 to inform the design of novel OA therapeutics.

Gastrodin is an emerging anti-inflammatory agent that has not been well studied within the context of OA. Nevertheless, gastrodin can inhibit chondrocyte senescence and mitochondrial damage in OA by regulating PI3K-AKT pathway phosphorylation via SIRT3 (Zhang et al., 2023). This suggests that gastrodin might represent an effective treatment option for OA and reinforces the importance of SIRT3 in mitochondrial homeostasis and its potential to regulate OA.

Methyl gallate is a plant-derived phenolic compound that has shown significant anti-inflammatory effects in different experimental models and potential applications for OA (Correa et al., 2022). The relationship between SIRT3 and methyl gallate has also been tentatively explored, revealing that methyl gallate treatment enhances mitochondrial autophagy by upregulating the expression of SIRT3, leading to anti-apoptotic and protective effects for extracellular matrix synthesis (Li et al., 2023b). These findings suggest that SIRT3 plays a positive role in OA therapies.

Mitochondrial acid-5 (4-(2,4-difluorophenyl)-2-(1H-indole-3-yl)-4-oxobutanoic acid; MA-5) is a mitochondrial homing drug that regulates mitochondrial energy metabolism by maintaining the membrane potential (Suzuki et al., 2015; Lei et al., 2018). SIRT3 is located upstream of Parkin, and MA-5 can protect chondrocytes by enhancing SIRT3 activity, promoting parkin-dependent mitochondrial autophagy, and eliminating depolarized/damaged mitochondria from chondrocytes (Xin et al., 2022).

SIRT3 may represent an emerging regulator of OA by modulating OA progression by regulating mitochondrial homeostasis. Although the more hierarchical mechanism of environment-dependent regulation of OA by SIRT3 is uncharacterized, SIRT3 and its upstream and downstream factors are of great value for treating OA. However, the role of SIRT3 and its upstream and downstream relationships require further investigation.

### 5.4 Femoral head necrosis

Femoral head necrosis is closely related to bone homeostasis (Liu et al., 2020; Nan et al., 2021). Glucocorticoids are a common clinical cause of osteonecrosis of the femoral head (GIONFH), typically resulting from long-term or excessive glucocorticoid intervention (Zheng et al., 2018). Glucocorticoid exposure leads to oxidative stress and promotes ROS overproduction, thereby damaging cell membranes and DNA, impairing osteogenic differentiation of BMSCs, and promoting apoptosis (Lee et al., 2017). In contrast, reducing the level of oxidative stress restores the stemness of BMSCs and prevents GIONFH development (Chen et al., 2019; Zhang et al., 2020b; Kubo et al., 2021). This suggests that inhibiting intracellular oxidative stress is key to treating GIONFH. Chen et al. found that SIRT3 expression is decreased in a rat model of GIONFH while resveratrol administration reduces the level of oxidative stress by enhancing SIRT3 expression and promoting the osteogenic differentiation of BMSCs, thereby delaying the GIONFH process (Chen et al., 2021). This suggests that SIRT3 may be a potential target for the treatment of GIONFH.

**FIGURE 5:**
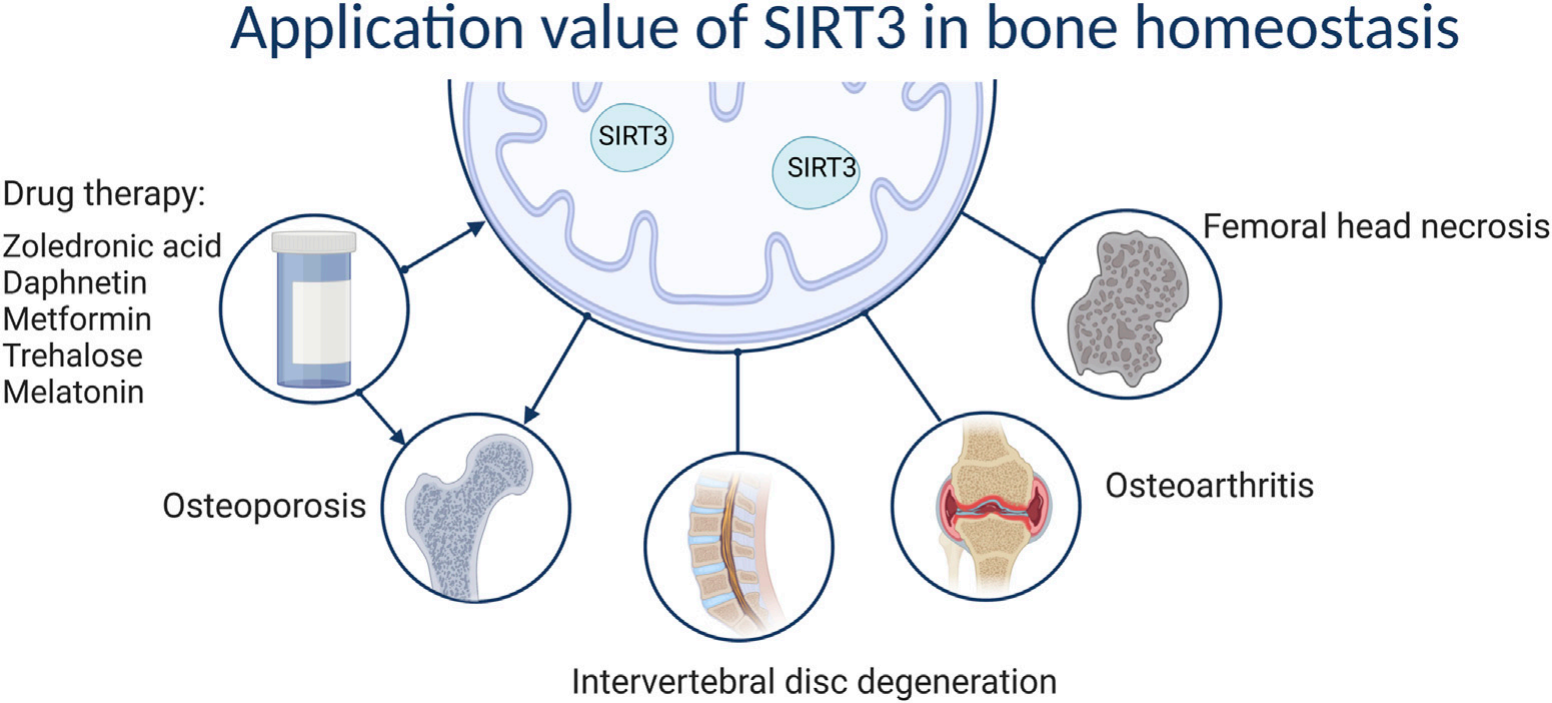
The value of SIRT3 in bone homeostasis.

SIRT3 plays a unique role in bone homeostasis-related diseases, such as OP and IDD. SIRT3 can prevent and delay OP and GIONFH by regulating bone homeostasis through anti-oxidative stress and regulating mitochondrial autophagy. Although SIRT3 delays IDD through anti-oxidative stress effects, whether SIRT3 can improve OP by regulating bone homeostasis and thereby improve IDD has not been determined. In OA, the role of SIRT3 is more complex and requires analyses on a case-by-case basis. Hence, the functions of SIRT3 in bone appear to be highly dependent on context. In particular, in OP development, although SIRT3 positively affects OB osteogenic differentiation, it promotes OC differentiation during the aging stage. Its complexity in regulating OP according to age, sex, and cell type may contribute to this outcome. Similarly, SIRT3 has specific effects on OA that may be related to the microenvironment of the organism, such as high lipid status, advanced age, and oxidative stress levels, among other factors. Nevertheless, although it is clear that SIRT3 has great potential for treating bone-related diseases, such as OP and IDD, further validation through additional animal studies and clinical trials is needed to provide new drug targets (Figure 5).

## 6 Activators/inhibitors of SIRT3

HKL is a pleiotropic compound isolated from *Magnolia* spp. (e.g. *Magnolia grandiflora* and *Magnolia alba*) that possesses potent antioxidant activity (Zhao and Liu, 2011). SIRT3 physically binds to HKL entering mitochondria via membrane diffusion (Zhang and Wang, 2010), and the bound SIRT3 has higher deacetylation activity, which increases mitochondrial biogenesis and activates MnSOD. Meanwhile, HKL is a potent activator of SIRT3 with various drug properties. For instance, administering a high dose (2 mg/kg/d) of HKL to aged mice (20 months) for 2 months leads to significant increases in SIRT3 expression, bone trabecular density, and bone volume to tissue ratio (Li et al., 2022c). In contrast, a low dose (0.2 mg/kg/d) of HKL has no such effect, suggesting that the beneficial effects of HKL on mouse bones are dose-dependent. Consistent with this effect, *in vitro* HKL treatment significantly increased SIRT3 expression in OBs. Notably, high doses of HKL not only prevent age-related bone loss but also increase OB responses to fluid shear stress *in vitro*. This effect is associated with a significant increase in SIRT3 expression, suggesting that HKL can activate SIRT3 to regulate bone homeostasis and improve bone health (Li et al., 2022c). This finding confirms that the SIRT3 activator promotes bone health while also enhancing the sensitivity of osteocytes to fluid shear stress *in vitro*. Hence, the combination of SIRT3 activator stimulation and exercise may amplify the beneficial effects of exercise to further promote bone health. In addition, HKL improves diabetic fracture healing by upregulating SIRT3 to promote oxidative stress resistance, thus suggesting a potential new therapeutic approach (Huang et al., 2022).

In contrast, LC-0296, a small molecule antagonist of SIRT3 synthesized *in vitro*, inhibits the enzymatic activity of SIRT3 with a selectivity approximately 20-fold higher than that for other SIRTs (Alhazzazi et al., 2016). Notably, intraperitoneal administration of the SIRT3 inhibitor LC-0296 (5 μg/g body weight, 100 μL/each) to 12-month-old female senescent mice for 4 months increased the thickness of the femoral cortex and slightly increased the amount of cancellous bone in the vertebrae (Ling et al., 2021). Consistent with this effect, the serum c-terminal peptide of type 1 collagen levels were significantly reduced in LC-0296-treated mice (Ling et al., 2021). Moreover, adding LC-0296 to OC bone progenitor cell cultures from 16-month-old female senescent mice resulted in effective OC formation and expression of late OC differentiation markers (Ling et al., 2021). These results indicate that LC-0296 intervention increases bone mass in aging mice by attenuating bone resorption. This finding is consistent with the conclusion that “SIRT3 contributes to the increase of OC bone resorption during skeletal aging,” suggesting that stimulating SIRT3 may be a good strategy to ameliorate metabolic bone diseases associated with aging.

In summary, HKL and LC-0296 activate and inhibit SIRT3 activity, respectively; however, their effects on bone homeostasis are consistent and positive, likely due to their different mechanisms of action. HKL has a dose-dependent effect on SIRT3; high doses promote SIRT3 expression in OBs, thereby ameliorating age-related bone turnover. Further exploration of the precise effector cells associated with HKL-regulated bone health is required. Meanwhile, LC-0296 positively affects bone by inhibiting bone resorption in aging mice. This discovery for HKL—a drug that already has clear clinical value—could provide novel insights for its repurposing. Moreover, the discovery of the medicinal value of LC-0296, which is a small molecule antagonist newly discovered in 2016, could help to develop new targeted therapies to improve bone health and promote quality of life in elderly OP patients. Future large-scale, multicenter, prospective clinical trials will validate SIRT3 drug development and the potential side effects and adverse effects of its inhibitors and activators, providing new directions for clinically preventing and treating bone homeostasis-related diseases and improving public health.

## 7 Effect of exercise on bone homeostasis via SIRT3

Exercise positively affects human health by reducing the risk of cardiovascular diseases, improving cardiorespiratory endurance, preventing OP, and eliciting anti-aging effects (Fiuza-Luces et al., 2018; Armstrong and Vogiatzis, 2019; Daly et al., 2019; Sujkowski et al., 2022). Exercise is strongly associated with bone health, and appropriate and regular exercise may help to prevent and improve OP (Howe et al., 2011a). However, a study in 2006 revealed that the effectiveness of exercise in counteracting bone fragility is markedly lower in middle-aged and older adults compared with young adults (Korpelainen et al., 2006; Howe et al., 2011a; Srinivasan et al., 2012); this may be primarily because of the reduced bone mineral density and decreased ability of bone cells to convert mechanical stimuli into biochemical signals observed in the older adults (Klein-Nulend et al., 2002; Busse et al., 2010). Therefore, strategies to improve the sensitivity of bones to mechanical stimuli in middle-aged and elderly populations and enhance the efficiency of exercise in preventing and mitigating bone loss must be developed. In 2022, Li et al. knocked down SIRT3 in MLO-Y4 cells and observed that the sensitivity of OB-like cells to fluid shear stress is significantly reduced compared with that in the control group (Li et al., 2022c). Similarly, running significantly increases the trabecular density and thickness and bone volume-to-tissue ratio in the femur/tibia of control mice compared with OB SIRT3-specific knockout mice. In contrast, no changes were observed in the cortical and cancellous bone parameters in the SIRT3-knockout group (Li et al., 2022c). This suggests that the anabolic effect of running on bone reconstruction is significantly attenuated in SIRT3-knockdown mice, while the blunted skeletal response to exercise in aged mice may be due to SIRT3 downregulation.

Relatively few studies have evaluated whether exercise regulates bone metabolism via SIRT3. Exercise regulates bone homeostasis by affecting the signature factors in bone; for example, exercise stimulates RUNX2, which is an essential transcription factor in regulating OB differentiation (Kanno et al., 2007), and modulates SIRT3. In human clinical trials, prolonged endurance training (≥8 weeks) increases SIRT3 expression in the serum and skeletal muscle of people of all ages. Meanwhile, resistance exercise increases SIRT3 levels in skeletal muscle in the older population. Moreover, in animal experiments, 8–12 weeks of endurance training increased SIRT3 activity in the brain, liver, and skeletal muscles (Zhou et al., 2022). Hence, regular exercise increases SIRT3 expression in various tissues and mediates SIRT3 metabolism and deacetylation to alleviate age-induced SIRT3 inhibition, thereby ameliorating aging-related diseases (Zhou et al., 2022).

In addition, irisin is a novel motor factor that is upregulated by exercise in various tissues/cells, serving as a powerful target for the treatment of various metabolic diseases (Boström et al., 2012). Under diabetic conditions, irisin can inhibit oxidative stress and mitochondrial autophagy through SIRT3 signaling and promote osteogenesis (Li et al., 2022a). This further demonstrates the important role of exercise in promoting bone metabolism by SIRT3.

Mechanistically, E11/gp38 protein expression is significantly reduced in the cortical bone of OB SIRT3-specific knockdown mice (Li et al., 2022c). Moreover, cAMP response element binding protein (CREB) might serve as a transcription factor linking SIRT3 and E11/gp38. Consistent with this effect, a significant reduction in E11/gp38 expression occurs in SIRT3-knockdown bone-like cells, accompanied by suppressed CREB phosphorylation (Li et al., 2022c). Given that CREB phosphorylates protein kinase A (PKA) (Alberini, 2009), PKA phosphorylation levels are also significantly reduced in SIRT3-knockdown bone-like cells. Activating the PKA-CREB signaling pathway using HKL restores the phenotype of SIRT3-deficient OBs and enhances OB sensitivity to fluid shear stress. Interestingly, PKA-CREB-E11/gp38 is a downstream factor in SIRT3-regulated dendritic cell processes in OBs (Li et al., 2022c). Hence, the PKA–CREB signaling pathway may mediate the regulatory effect of SIRT3 on E11/gp38 expression.

Although relatively few studies have focused on the exercise-mediated regulation of bone metabolism by SIRT3, indirect evidence suggests that exercise promotes SIRT3 upregulation in various tissues. SIRT3 then mediates the positive effect of exercise on bone health. Mechanistically, SIRT3 may regulate E11/gp38 through the PKA/CREB signaling pathway (Li et al., 2022c), thereby regulating the mechanical response to mediate bone mass and alleviating age-related bone homeostasis in bone disease. Hence, this represents a potentially novel mechanism by which exercise improves bone health and provides a new research direction for the clinical treatment of bone diseases. The close association between SIRT3 and exercise and their mutually reinforcing effects suggest that SIRT3 is likely an emerging exercise effector of considerable value in regulating bone health. However, the role of SIRT3 in regulating bone metabolism during different forms of exercise requires further exploration. Nevertheless, active and effective measures can be taken to explore the specific mechanisms underlying SIRT3-mediated exercise regulation of bone health and the role of SIRT3 in regulating bone homeostasis at all ages to prevent and improve bone metabolic diseases associated with aging.

## 8 Summary and outlook

Bone homeostasis is crucial for maintaining bone health and preventing and controlling bone diseases. As such, elucidating the mechanism(s) underlying bone homeostasis and identifying the associated targets will provide a theoretical basis for the clinical formulation of effective therapeutic regimens and drug therapies. Various novel cytokines have recently been identified as potential targets. One such cytokine is SIRT3, which participates in bone homeostasis via the differentiation and senescence of BMSCs, OBs, and OCs. However, SIRT3 acts in a highly context-dependent manner in bone and must be analyzed on a case-by-case basis. SIRT3 improves bone homeostasis-related diseases by responding to the mechanical stimuli generated by exercise. Notably, both SIRT3 activators and inhibitors can benefit bone, although the exact mechanisms underlying these effects require further exploration.

In bone diseases, especially OP, pharmacological treatment is currently divided into two main categories: bone-formation and bone-resorption inhibitors. However, most of these therapies have certain limitations. For instance, bisphosphonate treatment does not elicit therapeutic effects after 5 years of use. Moreover, most anti-OP drugs rapidly decline in efficacy once discontinued. Thus, developing safe, effective, and risk-free novel strategies to promote bone regeneration in OP patients has become a common research focus. Whether SIRT3 represents a potential therapeutic target for treating OP based on its bone resorption and formation effects also requires further evaluation. In addition, the mechanisms by which SIRT3 activators and inhibitors act on bone are currently unknown and require further exploration. Moreover, further studies are required to develop methods of combining SIRT3 with exercise to regulate bone mass and exploiting SIRT3 activators and inhibitors to develop novel therapeutics to treat bone metabolic diseases related to bone loss.

## Footnotes

1
https://www.who.int/news-room/fact-sheets/detail/musculoskeletal-conditions [Accessed 4 April 2023]

2
https://www.ncbi.nlm.nih.gov/pmc/articles/PMC9594975/[Accessed 31 January 2023]

3
https://www.ncbi.nlm.nih.gov/pmc/articles/PMC6232087/[Accessed 15 February 2023]

## References

[B1] Alberini C. M. (2009). Transcription factors in long-term memory and synaptic plasticity. Physiol. Rev. 89, 121–145. 10.1152/physrev.00017.2008 19126756 PMC3883056

[B2] Alhazzazi T. Y. Kamarajan P. Xu Y. Ai T. Chen L. Verdin E. (2016). A novel sirtuin-3 inhibitor, LC-0296, inhibits cell survival and proliferation, and promotes apoptosis of head and neck cancer cells. Anticancer Res. 36, 49–60. 26722027 PMC5417072

[B3] Ambrosi T. H. Scialdone A. Graja A. Gohlke S. Jank A.-M. Bocian C. (2017). Adipocyte accumulation in the bone marrow during obesity and aging impairs stem cell-based hematopoietic and bone regeneration. Cell. Stem Cell. 20, 771–784. 10.1016/j.stem.2017.02.009 28330582 PMC5459794

[B4] Anamika null Khanna A. Acharjee P. Acharjee A. Trigun S. K. (2019). Mitochondrial SIRT3 and neurodegenerative brain disorders. J. Chem. Neuroanat. 95, 43–53. 10.1016/j.jchemneu.2017.11.009 29129747

[B5] Arai C. Suyama A. Arai S. Arai N. Yoshizane C. Koya-Miyata S. (2020). Trehalose itself plays a critical role on lipid metabolism: trehalose increases jejunum cytoplasmic lipid droplets which negatively correlated with mesenteric adipocyte size in both HFD-fed trehalase KO and WT mice. Nutr. Metab. (Lond) 17, 22. 10.1186/s12986-020-00443-1 32206077 PMC7081596

[B6] Armstrong M. Vogiatzis I. (2019). Personalized exercise training in chronic lung diseases. Respirology 24, 854–862. 10.1111/resp.13639 31270909

[B7] Asumda F. Z. Chase P. B. (2012). Nuclear cardiac troponin and tropomyosin are expressed early in cardiac differentiation of rat mesenchymal stem cells. Differentiation 83, 106–115. 10.1016/j.diff.2011.10.002 22364878

[B8] Aventaggiato M. Barreca F. Vitiello L. Vespa S. Valente S. Rotili D. (2023). Role of SIRT3 in microgravity response: a new player in muscle tissue recovery. Cells 12, 691. 10.3390/cells12050691 36899828 PMC10000945

[B9] Boström P. Wu J. Jedrychowski M. P. Korde A. Ye L. Lo J. C. (2012). A PGC1-α-dependent myokine that drives brown-fat-like development of white fat and thermogenesis. Nature 481, 463–468. 10.1038/nature10777 22237023 PMC3522098

[B10] Busse B. Djonic D. Milovanovic P. Hahn M. Püschel K. Ritchie R. O. (2010). Decrease in the osteocyte lacunar density accompanied by hypermineralized lacunar occlusion reveals failure and delay of remodeling in aged human bone. Aging Cell. 9, 1065–1075. 10.1111/j.1474-9726.2010.00633.x 20874757

[B11] Cao L. Zhou S. Qiu X. Qiu S. (2022). Trehalose improves palmitic acid-induced apoptosis of osteoblasts by regulating SIRT3-medicated autophagy via the AMPK/mTOR/ULK1 pathway. FASEB J. 36, e22491. 10.1096/fj.202200608RR 35947089

[B12] Chen A. C. Martin A. J. Choy B. Fernández-Peñas P. Dalziell R. A. McKenzie C. A. (2015). A phase 3 randomized trial of nicotinamide for skin-cancer chemoprevention. N. Engl. J. Med. 373, 1618–1626. 10.1056/NEJMoa1506197 26488693

[B13] Chen D. Xia D. Pan Z. Xu D. Zhou Y. Wu Y. (2016). Metformin protects against apoptosis and senescence in nucleus pulposus cells and ameliorates disc degeneration *in vivo* . Cell. Death Dis. 7, e2441. 10.1038/cddis.2016.334 27787519 PMC5133996

[B14] Chen L. Wang B.-Z. Xie J. Zhang R.-Y. Jin C. Chen W.-K. (2021). Therapeutic effect of SIRT3 on glucocorticoid-induced osteonecrosis of the femoral head via intracellular oxidative suppression. Free Radic. Biol. Med. 176, 228–240. 10.1016/j.freeradbiomed.2021.07.016 34260898

[B15] Chen L.-Y. Wang Y. Terkeltaub R. Liu-Bryan R. (2018). Activation of AMPK-SIRT3 signaling is chondroprotective by preserving mitochondrial DNA integrity and function. Osteoarthr. Cartil. 26, 1539–1550. 10.1016/j.joca.2018.07.004 PMC6202232 30031925

[B16] Chen X.-J. Shen Y.-S. He M.-C. Yang F. Yang P. Pang F.-X. (2019). Polydatin promotes the osteogenic differentiation of human bone mesenchymal stem cells by activating the BMP2-Wnt/β-catenin signaling pathway. Biomed. Pharmacother. 112, 108746. 10.1016/j.biopha.2019.108746 30970530

[B17] Correa L. B. Pádua T. A. Alabarse P. V. G. Saraiva E. M. Garcia E. B. Amendoeira F. C. (2022). Protective effect of methyl gallate on murine antigen-induced arthritis by inhibiting inflammatory process and bone erosion. Inflammopharmacology 30, 251–266. 10.1007/s10787-021-00922-8 35112275

[B18] Croucher P. I. McDonald M. M. Martin T. J. (2016). Bone metastasis: the importance of the neighbourhood. Nat. Rev. Cancer 16, 373–386. 10.1038/nrc.2016.44 27220481

[B19] Da W. Tao L. Zhu Y. (2021). The role of osteoclast energy metabolism in the occurrence and development of osteoporosis. Front. Endocrinol. (Lausanne) 12, 675385. 10.3389/fendo.2021.675385 34054735 PMC8150001

[B20] Daly R. M. Dalla Via J. Duckham R. L. Fraser S. F. Helge E. W. (2019). Exercise for the prevention of osteoporosis in postmenopausal women: an evidence-based guide to the optimal prescription. Braz J. Phys. Ther. 23, 170–180. 10.1016/j.bjpt.2018.11.011 30503353 PMC6429007

[B21] Denu R. A. (2017). SIRT3 enhances mesenchymal stem cell longevity and differentiation. Oxid. Med. Cell. Longev. 2017, 5841716. 10.1155/2017/5841716 28717408 PMC5499245

[B22] Diao Z. Ji Q. Wu Z. Zhang W. Cai Y. Wang Z. (2021). SIRT3 consolidates heterochromatin and counteracts senescence. Nucleic Acids Res. 49, 4203–4219. 10.1093/nar/gkab161 33706382 PMC8096253

[B23] Ding Y. Yang H. Wang Y. Chen J. Ji Z. Sun H. (2017). Sirtuin 3 is required for osteogenic differentiation through maintenance of PGC-1ɑ-SOD2-mediated regulation of mitochondrial function. Int. J. Biol. Sci. 13, 254–264. 10.7150/ijbs.17053 28255277 PMC5332879

[B24] Domazetovic V. Marcucci G. Iantomasi T. Brandi M. L. Vincenzini M. T. (2017). Oxidative stress in bone remodeling: role of antioxidants. Clin. Cases Min. Bone Metab. 14, 209–216. 10.11138/ccmbm/2017.14.1.209 PMC5726212 29263736

[B25] Feng X. McDonald J. M. (2011). Disorders of bone remodeling. Annu. Rev. Pathol. 6, 121–145. 10.1146/annurev-pathol-011110-130203 20936937 PMC3571087

[B26] Fiuza-Luces C. Santos-Lozano A. Joyner M. Carrera-Bastos P. Picazo O. Zugaza J. L. (2018). Exercise benefits in cardiovascular disease: beyond attenuation of traditional risk factors. Nat. Rev. Cardiol. 15, 731–743. 10.1038/s41569-018-0065-1 30115967

[B27] Fu Y. Kinter M. Hudson J. Humphries K. M. Lane R. S. White J. R. (2016). Aging promotes sirtuin 3-dependent cartilage superoxide dismutase 2 acetylation and osteoarthritis. Arthritis Rheumatol. 68, 1887–1898. 10.1002/art.39618 26866626 PMC5331855

[B28] Gao J. Feng Z. Wang X. Zeng M. Liu J. Han S. (2018). SIRT3/SOD2 maintains osteoblast differentiation and bone formation by regulating mitochondrial stress. Cell. Death Differ. 25, 229–240. 10.1038/cdd.2017.144 28914882 PMC5762839

[B29] Gao J. Wang Z. Gao P. Fan Q. Zhang T. Cui L. (2022). Daphnetin alleviates senile and disuse osteoporosis by distinct modulations of bone formation and resorption. Antioxidants (Basel) 11, 2365. 10.3390/antiox11122365 36552574 PMC9774389

[B30] gianni (2019). SIRT3 retards intervertebral disc degeneration by anti-oxidative stress by activating the SIRT3/FOXO3/SOD2 signaling pathway. Eur. Rev. Available at: https://www.europeanreview.org/article/19408 (Accessed February 15, 2023). 10.26355/eurrev_201911_19408 31773668

[B31] gianni (2020). Zoledronic acid accelerates osteogenesis of bone marrow mesenchymal stem cells by attenuating oxidative stress via the SIRT3/SOD2 pathway and thus alleviates osteoporosis. European Review. Available at: https://www.europeanreview.org/article/20389 (Accessed January 31, 2023). 10.26355/eurrev_202002_20389 32141579

[B32] Giralt A. Hondares E. Villena J. A. Ribas F. Díaz-Delfín J. Giralt M. (2011). Peroxisome proliferator-activated receptor-gamma coactivator-1alpha controls transcription of the Sirt3 gene, an essential component of the thermogenic brown adipocyte phenotype. J. Biol. Chem. 286, 16958–16966. 10.1074/jbc.M110.202390 21454513 PMC3089539

[B33] Goodman S. B. (2007). Wear particles, periprosthetic osteolysis and the immune system. Biomaterials 28, 5044–5048. 10.1016/j.biomaterials.2007.06.035 17645943 PMC2065897

[B34] Guo J. Chiang W.-C. (2022). Mitophagy in aging and longevity. IUBMB Life 74, 296–316. 10.1002/iub.2585 34889504

[B35] Guo Y. Jia X. Cui Y. Song Y. Wang S. Geng Y. (2021). Sirt3-mediated mitophagy regulates AGEs-induced BMSCs senescence and senile osteoporosis. Redox Biol. 41, 101915. 10.1016/j.redox.2021.101915 33662874 PMC7930642

[B36] Han Y. You X. Xing W. Zhang Z. Zou W. (2018). Paracrine and endocrine actions of bone-the functions of secretory proteins from osteoblasts, osteocytes, and osteoclasts. Bone Res. 6, 16. 10.1038/s41413-018-0019-6 29844945 PMC5967329

[B37] Harada S. Rodan G. A. (2003). Control of osteoblast function and regulation of bone mass. Nature 423, 349–355. 10.1038/nature01660 12748654

[B38] He Y. Wu Z. Xu L. Xu K. Chen Z. Ran J. (2020). The role of SIRT3-mediated mitochondrial homeostasis in osteoarthritis. Cell. Mol. Life Sci. 77, 3729–3743. 10.1007/s00018-020-03497-9 32468094 PMC11105031

[B39] Hirschey M. D. Shimazu T. Goetzman E. Jing E. Schwer B. Lombard D. B. (2010). SIRT3 regulates mitochondrial fatty-acid oxidation by reversible enzyme deacetylation. Nature 464, 121–125. 10.1038/nature08778 20203611 PMC2841477

[B40] Hirschey M. D. Shimazu T. Jing E. Grueter C. A. Collins A. M. Aouizerat B. (2011). SIRT3 deficiency and mitochondrial protein hyperacetylation accelerate the development of the metabolic syndrome. Mol. Cell. 44, 177–190. 10.1016/j.molcel.2011.07.019 21856199 PMC3563434

[B41] Ho L. Wang L. Roth T. M. Pan Y. Verdin E. M. Hsiao E. C. (2017). Sirtuin-3 promotes adipogenesis, osteoclastogenesis, and bone loss in aging male mice. Endocrinology 158, 2741–2753. 10.1210/en.2016-1739 28911171 PMC5659662

[B42] Howe T. E. Shea B. Dawson L. J. Downie F. Murray A. Ross C. (2011a). Exercise for preventing and treating osteoporosis in postmenopausal women. Cochrane Database Syst. Rev., CD000333. 10.1002/14651858.CD000333.pub2 21735380 PMC12744941

[B43] Hu J. Kan T. Hu X. (2019). Sirt3 regulates mitophagy level to promote diabetic corneal epithelial wound healing. Exp. Eye Res. 181, 223–231. 10.1016/j.exer.2019.02.011 30794763

[B44] Hu S. Wang S. (2022). The role of SIRT3 in the osteoporosis. Front. Endocrinol. (Lausanne) 13, 893678. 10.3389/fendo.2022.893678 35692409 PMC9175005

[B45] Hu S. Zhang C. Qian T. Bai Y. Chen L. Chen J. (2021). Promoting nrf2/sirt3-dependent mitophagy suppresses apoptosis in nucleus pulposus cells and protects against intervertebral disc degeneration. Oxid. Med. Cell. Longev. 2021, 6694964. 10.1155/2021/6694964 34211633 PMC8211502

[B46] Huang X. Shu H. Ren C. Zhu J. (2022). SIRT3 improves bone regeneration and rescues diabetic fracture healing by regulating oxidative stress. Biochem. Biophys. Res. Commun. 604, 109–115. 10.1016/j.bbrc.2022.03.001 35303676

[B47] Hügle T. Geurts J. (2017). What drives osteoarthritis? synovial versus subchondral bone pathology. Rheumatol. Oxf. 56, 1461–1471. 10.1093/rheumatology/kew389 28003493

[B48] Huh J.-E. Shin J. H. Jang E. S. Park S. J. Park D. R. Ko R. (2016). Sirtuin 3 (SIRT3) maintains bone homeostasis by regulating AMPK-PGC-1β axis in mice. Sci. Rep. 6, 22511. 10.1038/srep22511 26928655 PMC4772385

[B49] Jing E. Emanuelli B. Hirschey M. D. Boucher J. Lee K. Y. Lombard D. (2011). Sirtuin-3 (Sirt3) regulates skeletal muscle metabolism and insulin signaling via altered mitochondrial oxidation and reactive oxygen species production. Proc. Natl. Acad. Sci. U. S. A. 108, 14608–14613. 10.1073/pnas.1111308108 21873205 PMC3167496

[B50] Kalinkovich A. Livshits G. (2021). Biased and allosteric modulation of bone cell-expressing G protein-coupled receptors as a novel approach to osteoporosis therapy. Pharmacol. Res. 171, 105794. 10.1016/j.phrs.2021.105794 34329703

[B51] Kanno T. Takahashi T. Tsujisawa T. Ariyoshi W. Nishihara T. (2007). Mechanical stress-mediated Runx2 activation is dependent on Ras/ERK1/2 MAPK signaling in osteoblasts. J. Cell. Biochem. 101, 1266–1277. 10.1002/jcb.21249 17265428

[B52] Kfoury Y. Scadden D. T. (2015). Mesenchymal cell contributions to the stem cell niche. Cell. Stem Cell. 16, 239–253. 10.1016/j.stem.2015.02.019 25748931

[B53] Khan N. M. Haseeb A. Ansari M. Y. Devarapalli P. Haynie S. Haqqi T. M. (2017). Wogonin, a plant derived small molecule, exerts potent anti-inflammatory and chondroprotective effects through the activation of ROS/ERK/Nrf2 signaling pathways in human Osteoarthritis chondrocytes. Free Radic. Biol. Med. 106, 288–301. 10.1016/j.freeradbiomed.2017.02.041 28237856 PMC5490997

[B54] Kim B.-S. Kim S.-J. Kim H.-J. Lee S.-J. Park Y.-J. Lee J. (2012). Effects of nicotine on proliferation and osteoblast differentiation in human alveolar bone marrow-derived mesenchymal stem cells. Life Sci. 90, 109–115. 10.1016/j.lfs.2011.10.019 22115820

[B55] Kim H. Lee Y. D. Kim H. J. Lee Z. H. Kim H.-H. (2017). SOD2 and Sirt3 control osteoclastogenesis by regulating mitochondrial ROS. J. Bone Min. Res. 32, 397–406. 10.1002/jbmr.2974 27540894

[B56] Kincaid B. Bossy-Wetzel E. (2013). Forever young: SIRT3 a shield against mitochondrial meltdown, aging, and neurodegeneration. Front. Aging Neurosci. 5, 48. 10.3389/fnagi.2013.00048 24046746 PMC3764375

[B57] Klein-Nulend J. Sterck J. G. H. Semeins C. M. Lips P. Joldersma M. Baart J. A. (2002). Donor age and mechanosensitivity of human bone cells. Osteoporos. Int. 13, 137–146. 10.1007/s001980200005 11908490

[B58] Korpelainen R. Keinänen-Kiukaanniemi S. Heikkinen J. Väänänen K. Korpelainen J. (2006). Effect of impact exercise on bone mineral density in elderly women with low BMD: a population-based randomized controlled 30-month intervention. Osteoporos. Int. 17, 109–118. 10.1007/s00198-005-1924-2 15889312

[B59] Kubo Y. Drescher W. Fragoulis A. Tohidnezhad M. Jahr H. Gatz M. (2021). Adverse effects of oxidative stress on bone and vasculature in corticosteroid-associated osteonecrosis: potential role of nuclear factor erythroid 2-related factor 2 in cytoprotection. Antioxid. Redox Signal 35, 357–376. 10.1089/ars.2020.8163 33678001

[B60] Lane J. M. Russell L. Khan S. N. (2000). Osteoporosis. Clin. Orthop. Relat. Res. 372, 139–150. 10.1097/00003086-200003000-00016 10738423

[B61] Lee W.-C. Guntur A. R. Long F. Rosen C. J. (2017). Energy metabolism of the osteoblast: implications for osteoporosis. Endocr. Rev. 38, 255–266. 10.1210/er.2017-00064 28472361 PMC5460680

[B62] Lei Q. Tan J. Yi S. Wu N. Wang Y. Wu H. (2018). Mitochonic acid 5 activates the MAPK-ERK-yap signaling pathways to protect mouse microglial BV-2 cells against TNFα-induced apoptosis via increased Bnip3-related mitophagy. Cell. Mol. Biol. Lett. 23, 14. 10.1186/s11658-018-0081-5 29636771 PMC5887257

[B63] Li G. Jian Z. Wang H. Xu L. Zhang T. Song J. (2022a). Irisin promotes osteogenesis by modulating oxidative stress and mitophagy through SIRT3 signaling under diabetic conditions. Oxid. Med. Cell. Longev. 2022, 3319056. 10.1155/2022/3319056 36262283 PMC9576424

[B64] Li M. Yu Y. Xue K. Li J. Son G. Wang J. (2023a). Genistein mitigates senescence of bone marrow mesenchymal stem cells via ERRα-mediated mitochondrial biogenesis and mitophagy in ovariectomized rats. Redox Biol. 61, 102649. 10.1016/j.redox.2023.102649 36871183 PMC9995482

[B65] Li N. Li X. Zheng K. Bai J. Zhang W. Sun H. (2021a). Inhibition of Sirtuin 3 prevents titanium particle-induced bone resorption and osteoclastsogenesis via suppressing ERK and JNK signaling. Int. J. Biol. Sci. 17, 1382–1394. 10.7150/ijbs.53992 33867853 PMC8040473

[B66] Li Q. Cheng J. C. Jiang Q. Lee W. Y. (2021b). Role of sirtuins in bone biology: potential implications for novel therapeutic strategies for osteoporosis. Aging Cell. 20, e13301. 10.1111/acel.13301 33393735 PMC7884050

[B67] Li Q. Wang H. Zhang J. Kong A. P.-S. Li G. Lam T.-P. (2021c). Deletion of SIRT3 inhibits osteoclastogenesis and alleviates aging or estrogen deficiency-induced bone loss in female mice. Bone 144, 115827. 10.1016/j.bone.2020.115827 33359008

[B68] Li Q. Wang R. Zhang Z. Wang H. Lu X. Zhang J. (2022c). Sirt3 mediates the benefits of exercise on bone in aged mice. Cell. Death Differ. 30, 152–167. 10.1038/s41418-022-01053-5 36153410 PMC9883264

[B69] Li Y. Shen B. Lv C. Zhu X. Naren Q. Xu D. (2023b). Methyl gallate prevents oxidative stress induced apoptosis and ECM degradation in chondrocytes via restoring Sirt3 mediated autophagy and ameliorates osteoarthritis progression. Int. Immunopharmacol. 114, 109489. 10.1016/j.intimp.2022.109489 36459925

[B70] Li Y. Yu C. Shen G. Li G. Shen J. Xu Y. (2015). Sirt3-MnSOD axis represses nicotine-induced mitochondrial oxidative stress and mtDNA damage in osteoblasts. Acta Biochim. Biophys. Sin. (Shanghai) 47, 306–312. 10.1093/abbs/gmv013 25757953

[B71] Lin J. Du J. Wu X. Xu C. Liu J. Jiang L. (2021). SIRT3 mitigates intervertebral disc degeneration by delaying oxidative stress-induced senescence of nucleus pulposus cells. J. Cell. Physiol. 236, 6441–6456. 10.1002/jcp.30319 33565085

[B72] Ling W. Krager K. Richardson K. K. Warren A. D. Ponte F. Aykin-Burns N. (2021). Mitochondrial Sirt3 contributes to the bone loss caused by aging or estrogen deficiency. JCI Insight 6, 146728. 10.1172/jci.insight.146728 33878033 PMC8262324

[B73] Liu F. Yuan L. Li L. Yang J. Liu J. Chen Y. (2023). S-sulfhydration of SIRT3 combats BMSC senescence and ameliorates osteoporosis via stabilizing heterochromatic and mitochondrial homeostasis. Pharmacol. Res. 192, 106788. 10.1016/j.phrs.2023.106788 37146925

[B74] Liu H.-D. Ren M.-X. Li Y. Zhang R.-T. Ma N.-F. Li T.-L. (2022). Melatonin alleviates hydrogen peroxide induced oxidative damage in MC3T3-E1 cells and promotes osteogenesis by activating SIRT1. Free Radic. Res. 56, 63–76. 10.1080/10715762.2022.2037580 35109721

[B75] Liu K. Wang K. Wang L. Zhou Z. (2020). Changes of lipid and bone metabolism in broilers with spontaneous femoral head necrosis. Poult. Sci. 100, 100808. 10.1016/j.psj.2020.10.062 33518301 PMC7936160

[B76] Liu P. Zou D. Chen K. Zhou Q. Gao Y. Huang Y. (2016). Dihydromyricetin improves hypobaric hypoxia-induced memory impairment via modulation of SIRT3 signaling. Mol. Neurobiol. 53, 7200–7212. 10.1007/s12035-015-9627-y 26687185

[B77] Liu X. Gao X. Liu Y. Liang D. Fu T. Song Y. (2019). Daphnetin inhibits RANKL-induced osteoclastogenesis *in vitro* . J. Cell. Biochem. 120, 2304–2312. 10.1002/jcb.27555 30206967

[B78] Long F. (2011). Building strong bones: molecular regulation of the osteoblast lineage. Nat. Rev. Mol. Cell. Biol. 13, 27–38. 10.1038/nrm3254 22189423

[B79] Ma C. Sun Y. Pi C. Wang H. Sun H. Yu X. (2020). Sirt3 attenuates oxidative stress damage and rescues cellular senescence in rat bone marrow mesenchymal stem cells by targeting superoxide dismutase 2. Front. Cell. Dev. Biol. 8, 599376. 10.3389/fcell.2020.599376 33330487 PMC7718008

[B80] MacDonald I. J. Tsai H.-C. Chang A.-C. Huang C.-C. Yang S.-F. Tang C.-H. (2021). Melatonin inhibits osteoclastogenesis and osteolytic bone metastasis: implications for osteoporosis. Int. J. Mol. Sci. 22, 9435. 10.3390/ijms22179435 34502344 PMC8430520

[B81] Malemud C. J. (2017). “Chapter seven - matrix metalloproteinases and synovial joint pathology,” in *Progress in molecular Biology and translational science* matrix metalloproteinases and tissue remodeling in health and disease: target tissues and therapy. Editor Khalil R. A. China (Academic Press), 305–325. 10.1016/bs.pmbts.2017.03.003 28662824

[B82] Maneiro E. Martín M. A. de Andres M. C. López-Armada M. J. Fernández-Sueiro J. L. del Hoyo P. (2003). Mitochondrial respiratory activity is altered in osteoarthritic human articular chondrocytes. Arthritis Rheum. 48, 700–708. 10.1002/art.10837 12632423

[B83] Maruotti N. Corrado A. Cantatore F. P. (2017). Osteoblast role in osteoarthritis pathogenesis. J. Cell. Physiol. 232, 2957–2963. 10.1002/jcp.25969 28425564 PMC5575507

[B84] McArdle A. Marecic O. Tevlin R. Walmsley G. G. Chan C. K. F. Longaker M. T. (2015). The role and regulation of osteoclasts in normal bone homeostasis and in response to injury. Plast. Reconstr. Surg. 135, 808–816. 10.1097/PRS.0000000000000963 25719699

[B85] McDonnell E. Peterson B. S. Bomze H. M. Hirschey M. D. (2015). SIRT3 regulates progression and development of diseases of aging. Trends Endocrinol. Metab. 26, 486–492. 10.1016/j.tem.2015.06.001 26138757 PMC4558250

[B86] Mizoguchi T. Ono N. (2021). The diverse origin of bone-forming osteoblasts. J. Bone Min. Res. 36, 1432–1447. 10.1002/jbmr.4410 PMC8338797 34213032

[B87] Moerman E. J. Teng K. Lipschitz D. A. Lecka-Czernik B. (2004). Aging activates adipogenic and suppresses osteogenic programs in mesenchymal marrow stroma/stem cells: the role of PPAR-gamma2 transcription factor and TGF-beta/BMP signaling pathways. Aging Cell. 3, 379–389. 10.1111/j.1474-9728.2004.00127.x 15569355 PMC1850101

[B88] Nan K. Pei J. Fan L. Zhang Y. Zhang X. Liu K. (2021). Resveratrol prevents steroid-induced osteonecrosis of the femoral head via miR-146a modulation. Ann. N. Y. Acad. Sci. 1503, 23–37. 10.1111/nyas.14555 33454992

[B89] Nguyen G. T. T. Schaefer S. Gertz M. Weyand M. Steegborn C. (2013). Structures of human sirtuin 3 complexes with ADP-ribose and with carba-NAD+ and SRT1720: binding details and inhibition mechanism. Acta Crystallogr. D. Biol. Crystallogr. 69, 1423–1432. 10.1107/S0907444913015448 23897466

[B90] oxidative (2023). Total oxidative/anti-oxidative status and relation to bone mineral density in osteoporosis | SpringerLink. Available at: https://link.springer.com/article/10.1007/s00296-007-0452-0 (Accessed August 3, 2023). 10.1007/s00296-007-0452-0 17823800

[B91] Qadir A. Liang S. Wu Z. Chen Z. Hu L. Qian A. (2020). Senile osteoporosis: the involvement of differentiation and senescence of bone marrow stromal cells. Int. J. Mol. Sci. 21, 349. 10.3390/ijms21010349 31948061 PMC6981793

[B92] Reactive Oxygen (2023). Reactive oxygen species in osteoclast differentiation and possible pharmaceutical targets of ROS-mediated osteoclast diseases - PubMed. Available at: https://pubmed.ncbi.nlm.nih.gov/31336616/(Accessed August 3, 2023). 10.3390/ijms20143576 PMC6678498 31336616

[B93] Richardson K. K. Ling W. Krager K. Fu Q. Byrum S. D. Pathak R. (2022). Ionizing radiation activates mitochondrial function in osteoclasts and causes bone loss in young adult male mice. Int. J. Mol. Sci. 23, 675. 10.3390/ijms23020675 35054859 PMC8775597

[B94] Roodman G. D. (1996). Advances in bone biology: the osteoclast. Endocr. Rev. 17, 308–332. 10.1210/edrv-17-4-308 8854048

[B95] Saul D. Kosinsky R. L. (2021). Epigenetics of aging and aging-associated diseases. Int. J. Mol. Sci. 22, 401. 10.3390/ijms22010401 33401659 PMC7794926

[B96] Song Y. Li S. Geng W. Luo R. Liu W. Tu J. (2018). Sirtuin 3-dependent mitochondrial redox homeostasis protects against AGEs-induced intervertebral disc degeneration. Redox Biol. 19, 339–353. 10.1016/j.redox.2018.09.006 30216853 PMC6139007

[B97] Srinivasan S. Gross T. S. Bain S. D. (2012). Bone mechanotransduction may require augmentation in order to strengthen the senescent skeleton. Ageing Res. Rev. 11, 353–360. 10.1016/j.arr.2011.12.007 22240208 PMC3350758

[B98] Srivastava S. (2016). Emerging therapeutic roles for NAD(+) metabolism in mitochondrial and age-related disorders. Clin. Transl. Med. 5, 25. 10.1186/s40169-016-0104-7 27465020 PMC4963347

[B99] Srivastava S. P. Li J. Kitada M. Fujita H. Yamada Y. Goodwin J. E. (2018). SIRT3 deficiency leads to induction of abnormal glycolysis in diabetic kidney with fibrosis. Cell. Death Dis. 9, 997. 10.1038/s41419-018-1057-0 30250024 PMC6155322

[B100] Sujkowski A. L. Hong L. Wessells R. J. Todi S. V. (2022). The protective role of exercise against age-related neurodegeneration. Ageing Res. Rev. 74, 101543. 10.1016/j.arr.2021.101543 34923167 PMC8761166

[B101] Sun Q. Tian F.-M. Liu F. Fang J.-K. Hu Y.-P. Lian Q.-Q. (2021). Denosumab alleviates intervertebral disc degeneration adjacent to lumbar fusion by inhibiting endplate osteochondral remodeling and vertebral osteoporosis in ovariectomized rats. Arthritis Res. Ther. 23, 152. 10.1186/s13075-021-02525-8 34049577 PMC8161944

[B102] Sun W. Liu C. Chen Q. Liu N. Yan Y. Liu B. (2018). SIRT3: a new regulator of cardiovascular diseases. Oxid. Med. Cell. Longev. 2018, 7293861. 10.1155/2018/7293861 29643974 PMC5831850

[B103] Sundaresan N. R. Samant S. A. Pillai V. B. Rajamohan S. B. Gupta M. P. (2008). SIRT3 is a stress-responsive deacetylase in cardiomyocytes that protects cells from stress-mediated cell death by deacetylation of Ku70. Mol. Cell. Biol. 28, 6384–6401. 10.1128/MCB.00426-08 18710944 PMC2577434

[B104] Suzuki T. Yamaguchi H. Kikusato M. Matsuhashi T. Matsuo A. Sato T. (2015). Mitochonic acid 5 (MA-5), a derivative of the plant hormone indole-3-acetic acid, improves survival of fibroblasts from patients with mitochondrial diseases. Tohoku J. Exp. Med. 236, 225–232. 10.1620/tjem.236.225 26118651

[B105] Tabara Y. Ikezoe T. Yamanaka M. Setoh K. Segawa H. Kawaguchi T. (2019). Advanced glycation end product accumulation is associated with low skeletal muscle mass, weak muscle strength, and reduced bone density: the nagahama study. J. Gerontol. A Biol. Sci. Med. Sci. 74, 1446–1453. 10.1093/gerona/gly233 30329028

[B106] Tsai P.-H. Chien Y. Chuang J.-H. Chou S.-J. Chien C.-H. Lai Y.-H. (2015). Dysregulation of mitochondrial functions and osteogenic differentiation in cisd2-deficient murine induced pluripotent stem cells. Stem Cells Dev. 24, 2561–2576. 10.1089/scd.2015.0066 26230298 PMC4620525

[B107] Wang B. Zhan Y. Yan L. Hao D. (2022). How zoledronic acid improves osteoporosis by acting on osteoclasts. Front. Pharmacol. 13, 961941. 10.3389/fphar.2022.961941 36091799 PMC9452720

[B108] Wang C. Wang Y. Shen L. (2021). Mitochondrial proteins in heart failure: the role of deacetylation by SIRT3. Pharmacol. Res. 172, 105802. 10.1016/j.phrs.2021.105802 34363948

[B109] Wang J. Wang K. Huang C. Lin D. Zhou Y. Wu Y. (2018). SIRT3 activation by dihydromyricetin suppresses chondrocytes degeneration via maintaining mitochondrial homeostasis. Int. J. Biol. Sci. 14, 1873–1882. 10.7150/ijbs.27746 30443190 PMC6231225

[B110] Wang L. Qi H. Tang Y. Shen H.-M. (2020a). Post-translational modifications of key machinery in the control of mitophagy. Trends Biochem. Sci. 45, 58–75. 10.1016/j.tibs.2019.08.002 31606339

[B111] Wang L. You X. Lotinun S. Zhang L. Wu N. Zou W. (2020b). Mechanical sensing protein PIEZO1 regulates bone homeostasis via osteoblast-osteoclast crosstalk. Nat. Commun. 11, 282. 10.1038/s41467-019-14146-6 31941964 PMC6962448

[B112] Wang S. Yang J. Lin T. Huang S. Ma J. Xu X. (2020c). Excessive production of mitochondrion-derived reactive oxygen species induced by titanium ions leads to autophagic cell death of osteoblasts via the SIRT3/SOD2 pathway. Mol. Med. Rep. 22, 257–264. 10.3892/mmr.2020.11094 32468046 PMC7248520

[B113] Wang X. Liang T. Zhu Y. Qiu J. Qiu X. Lian C. (2019). Melatonin prevents bone destruction in mice with retinoic acid–induced osteoporosis. Mol. Med. 25, 43. 10.1186/s10020-019-0107-0 31462213 PMC6714316

[B114] Wang X.-Q. Shao Y. Ma C.-Y. Chen W. Sun L. Liu W. (2014). Decreased SIRT3 in aged human mesenchymal stromal/stem cells increases cellular susceptibility to oxidative stress. J. Cell. Mol. Med. 18, 2298–2310. 10.1111/jcmm.12395 25210848 PMC4224562

[B115] Wang Y. Chen J. Chen J. Dong C. Yan X. Zhu Z. (2020d). Daphnetin ameliorates glucocorticoid-induced osteoporosis via activation of Wnt/GSK-3β/β-catenin signaling. Toxicol. Appl. Pharmacol. 409, 115333. 10.1016/j.taap.2020.115333 33171191

[B116] Willey J. S. Lloyd S. A. J. Nelson G. A. Bateman T. A. (2011). Space radiation and bone loss. Gravit. Space Biol. Bull. 25, 14–21. 10.26355/eurrev_201911_19408 22826632 PMC3401484

[B117] Williams D. Kuipers A. Mukai C. Thirsk R. (2009). Acclimation during space flight: effects on human physiology. CMAJ 180, 1317–1323. 10.1503/cmaj.090628 19509005 PMC2696527

[B118] Xin R. Xu Y. Long D. Mao G. Liao H. Zhang Z. (2022). Mitochonic acid-5 inhibits reactive oxygen species production and improves human chondrocyte survival by upregulating SIRT3-mediated, parkin-dependent mitophagy. Front. Pharmacol. 13, 911716. 10.3389/fphar.2022.911716 35734404 PMC9207248

[B119] Xu K. He Y. Moqbel S. A. A. Zhou X. Wu L. Bao J. (2021). SIRT3 ameliorates osteoarthritis via regulating chondrocyte autophagy and apoptosis through the PI3K/Akt/mTOR pathway. Int. J. Biol. Macromol. 175, 351–360. 10.1016/j.ijbiomac.2021.02.029 33556400

[B120] Xu X. Wang R. Wu R. Yan W. Shi T. Jiang Q. (2020). Trehalose reduces bone loss in experimental biliary cirrhosis rats via ERK phosphorylation regulation by enhancing autophagosome formation. FASEB J. 34, 8402–8415. 10.1096/fj.201902528RRR 32367591

[B121] Yang K. Pei L. Zhou S. Tao L. Zhu Y. (2021). Metformin attenuates H2O2-induced osteoblast apoptosis by regulating SIRT3 via the PI3K/AKT pathway. Exp. Ther. Med. 22, 1316. 10.3892/etm.2021.10751 34630670 PMC8495548

[B122] Yang W. Nagasawa K. Münch C. Xu Y. Satterstrom K. Jeong S. (2016). Mitochondrial sirtuin network reveals dynamic SIRT3-dependent deacetylation in response to membrane depolarization. Cell. 167, 985–1000. 10.1016/j.cell.2016.10.016 27881304 PMC5134900

[B123] Yoon H. Park S. G. Kim H.-J. Shin H.-R. Kim K.-T. Cho Y.-D. (2023). Nicotinamide enhances osteoblast differentiation through activation of the mitochondrial antioxidant defense system. Exp. Mol. Med. 55, 1531–1543. 10.1038/s12276-023-01041-w 37464093 PMC10393969

[B124] Zaidi M. (2007). Skeletal remodeling in health and disease. Nat. Med. 13, 791–801. 10.1038/nm1593 17618270

[B125] Zhang D. Zhang C. Fu B. Sun L. Wang X. Chen W. (2018). Sirtuin3 protects aged human mesenchymal stem cells against oxidative stress and enhances efficacy of cell therapy for ischaemic heart diseases. J. Cell. Mol. Med. 22, 5504–5517. 10.1111/jcmm.13821 30091830 PMC6201360

[B126] Zhang D.-Y. Gao T. Xu R.-J. Sun L. Zhang C.-F. Bai L. (2020a). SIRT3 transfection of aged human bone marrow-derived mesenchymal stem cells improves cell therapy-mediated myocardial repair. Rejuvenation Res. 23, 453–464. 10.1089/rej.2019.2260 32228121

[B127] Zhang F. Peng W. Zhang J. Dong W. Wu J. Wang T. (2020b). P53 and Parkin co-regulate mitophagy in bone marrow mesenchymal stem cells to promote the repair of early steroid-induced osteonecrosis of the femoral head. Cell. Death Dis. 11, 42. 10.1038/s41419-020-2238-1 31959744 PMC6971291

[B128] Zhang G.-Z. Deng Y.-J. Xie Q.-Q. Ren E.-H. Ma Z.-J. He X.-G. (2020c). Sirtuins and intervertebral disc degeneration: roles in inflammation, oxidative stress, and mitochondrial function. Clin. Chim. Acta 508, 33–42. 10.1016/j.cca.2020.04.016 32348785

[B129] Zhang J. Xiang H. Liu J. Chen Y. He R.-R. Liu B. (2020d). Mitochondrial Sirtuin 3: new emerging biological function and therapeutic target. Theranostics 10, 8315–8342. 10.7150/thno.45922 32724473 PMC7381741

[B130] Zhang L. Wang X. (2010). Hydrophobic ionic liquid-based ultrasound-assisted extraction of magnolol and honokiol from cortex Magnoliae officinalis. J. Sep. Sci. 33, 2035–2038. 10.1002/jssc.201000076 20512806

[B131] Zhang Y. Jin W. Chen J. Wei S. Cai W. Zhong Y. (2023). Gastrodin alleviates rat chondrocyte senescence and mitochondrial dysfunction through Sirt3. Int. Immunopharmacol. 118, 110022. 10.1016/j.intimp.2023.110022 36933487

[B132] Zhao C. Liu Z.-Q. (2011). Comparison of antioxidant abilities of magnolol and honokiol to scavenge radicals and to protect DNA. Biochimie 93, 1755–1760. 10.1016/j.biochi.2011.06.012 21704114

[B133] Zheng K. Bai J. Li N. Li M. Sun H. Zhang W. (2021). Protective effects of sirtuin 3 on titanium particle-induced osteogenic inhibition by regulating the NLRP3 inflammasome via the GSK-3β/β-catenin signalling pathway. Bioact. Mater 6, 3343–3357. 10.1016/j.bioactmat.2021.02.039 33817415 PMC8005659

[B134] Zheng L.-Z. Wang J.-L. Kong L. Huang L. Tian L. Pang Q.-Q. (2018). Steroid-associated osteonecrosis animal model in rats. J. Orthop. Transl. 13, 13–24. 10.1016/j.jot.2018.01.003 PMC5892381 29662787

[B135] Zhou B. O. Yue R. Murphy M. M. Peyer J. G. Morrison S. J. (2014a). Leptin-receptor-expressing mesenchymal stromal cells represent the main source of bone formed by adult bone marrow. Cell. Stem Cell. 15, 154–168. 10.1016/j.stem.2014.06.008 24953181 PMC4127103

[B136] Zhou L. Pinho R. Gu Y. Radak Z. (2022). The role of SIRT3 in exercise and aging. Cells 11, 2596. 10.3390/cells11162596 36010672 PMC9406297

[B137] Zhou S. Greenberger J. S. Epperly M. W. Goff J. P. Adler C. Leboff M. S. (2008). Age-related intrinsic changes in human bone-marrow-derived mesenchymal stem cells and their differentiation to osteoblasts. Aging Cell. 7, 335–343. 10.1111/j.1474-9726.2008.00377.x 18248663 PMC2398731

[B138] Zhou W. Liu Y. Shen J. Yu B. Bai J. Lin J. (2019). Melatonin increases bone mass around the prostheses of OVX rats by ameliorating mitochondrial oxidative stress via the SIRT3/SOD2 signaling pathway. Oxid. Med. Cell. Longev. 2019, 4019619. 10.1155/2019/4019619 31110599 PMC6487111

[B139] Zhou Y. Liang X. Chang H. Shu F. Wu Y. Zhang T. (2014b). Ampelopsin-induced autophagy protects breast cancer cells from apoptosis through Akt-mTOR pathway via endoplasmic reticulum stress. Cancer Sci. 105, 1279–1287. 10.1111/cas.12494 25088800 PMC4462353

[B140] Zhu C. Shen S. Zhang S. Huang M. Zhang L. Chen X. (2022a). Autophagy in bone remodeling: a regulator of oxidative stress. Front. Endocrinol. (Lausanne) 13, 898634. 10.3389/fendo.2022.898634 35846332 PMC9279723

[B141] Zhu S. Donovan E. L. Makosa D. Mehta-D’souza P. Jopkiewicz A. Batushansky A. (2022b). Sirt3 promotes chondrogenesis, chondrocyte mitochondrial respiration and the development of high-fat diet-induced osteoarthritis in mice. J. Bone Mineral Res. 37, 2531–2547. 10.1002/jbmr.4721 PMC10091721 36214465

